# Clinically relevant antimicrobial resistance at the wildlife–livestock–human interface in Nairobi: an epidemiological study

**DOI:** 10.1016/S2542-5196(19)30083-X

**Published:** 2019-06

**Authors:** James M Hassell, Melissa J Ward, Dishon Muloi, Judy M Bettridge, Timothy P Robinson, Sam Kariuki, Allan Ogendo, John Kiiru, Titus Imboma, Erastus K Kang'ethe, Elin M Öghren, Nicola J Williams, Michael Begon, Mark E J Woolhouse, Eric M Fèvre

**Affiliations:** a Institute of Infection and Global Health, University of Liverpool, Neston, UK; b International Livestock Research Institute, Nairobi, Kenya; c Centre for Immunity, Infection and Evolution, University of Edinburgh, Edinburgh, UK; d Usher Institute of Population Health Sciences and Informatics, University of Edinburgh, Edinburgh, UK; e Nuffield Department of Clinical Medicine, University of Oxford, John Radcliffe Hospital, Oxford, UK; f Food and Agriculture Organization of the United Nations, Rome, Italy; g Centre for Microbiology Research, Kenya Medical Research Institute, Nairobi, Kenya; h National Museums of Kenya, Nairobi, Kenya; i University of Nairobi, Nairobi, Kenya; j Department of Medical Biochemistry and Microbiology, Uppsala University, Uppsala, Sweden; k Institute of Integrative Biology, University of Liverpool, Liverpool, UK

## Abstract

**Background:**

Antimicrobial resistance is one of the great challenges facing global health security in the modern era. Wildlife, particularly those that use urban environments, are an important but understudied component of epidemiology of antimicrobial resistance. We investigated antimicrobial resistance overlap between sympatric wildlife, humans, livestock, and their shared environment across the developing city of Nairobi, Kenya. We use these data to examine the role of urban wildlife in the spread of clinically relevant antimicrobial resistance.

**Methods:**

99 households across Nairobi were randomly selected on the basis of socioeconomic stratification. A detailed survey was administered to household occupants, and samples (n=2102) were collected from the faeces of 75 wildlife species inhabiting household compounds (ie, the household and its perimeter; n=849), 13 livestock species (n=656), and humans (n=333), and from the external environment (n=288). *Escherichia coli*, our sentinel organism, was cultured and a single isolate from each sample tested for sensitivity to 13 antibiotics. Diversity of antimicrobial resistant phenotypes was compared between urban wildlife, humans, livestock, and the environment, to investigate whether wildlife are a net source for antimicrobial resistance in Nairobi. Generalised linear mixed models were used to determine whether the prevalence of antimicrobial resistant phenotypes and multidrug-resistant *E coli* carriage in urban wildlife is linked to variation in ecological traits, such as foraging behaviour, and to determine household-level risk factors for sharing of antimicrobial resistance between humans, wildlife, and livestock.

**Findings:**

*E coli* were isolated from 485 samples collected from wildlife between Sept 6,2015, and Sept 28, 2016. Wildlife carried a low prevalence of *E coli* isolates susceptible to all antibiotics tested (45 [9%] of 485 samples) and a high prevalence of clinically relevant multidrug resistance (252 [52%] of 485 samples), which varied between taxa and by foraging traits. Multiple isolates were resistant to one agent from at least seven antimicrobial classes tested for, and a single isolate was resistant to all antibiotics tested for in the study. The phenotypic diversity of antimicrobial-resistant *E coli* in wildlife was lower than in livestock, humans, and the environment. Within household compounds, statistical models identified two interfaces for exchange of antimicrobial resistance: between both rodents, humans and their rubbish, and seed-eating birds, humans and their rubbish; and between seed-eating birds, cattle, and bovine manure.

**Interpretation:**

Urban wildlife carry a high burden of clinically relevant antimicrobial-resistant *E coli* in Nairobi, exhibiting resistance to drugs considered crucial for human medicine by WHO. Identifiable traits of the wildlife contribute to this exposure; however, compared with humans, livestock, and the environment, low phenotypic diversity in wildlife is consistent with the hypothesis that wildlife are a net sink rather than source of clinically relevant resistance. Wildlife that interact closely with humans, livestock, and both human and livestock waste within households, are exposed to more antimicrobial resistant phenotypes, and could therefore act as conduits for the dissemination of clinically relevant antimicrobial resistance to the wider environment. These results provide novel insight into the broader epidemiology of antimicrobial resistance in complex urban environments, characteristic of lower-middle-income countries.

**Funding:**

UK Medical Research Council and CGIAR Research Program on Agriculture for Nutrition and Health.

## Introduction

Antimicrobial resistance in bacteria is one of the great challenges facing global health security in the modern era, and will ultimately limit our capacity to treat microbial infections. The repercussions for human and domestic animal health are severe; as infections become more difficult and costly to treat, morbidity and mortality will increase, and the extra burden placed on health services and livestock production will have considerable economic consequences.^1^

The two most probable sources of clinically relevant antimicrobial resistance are the exposure of pathogens to antibiotic use in humans and in livestock.^2^ Little is known about the ecology of antimicrobial resistance outside human and livestock hosts, but it is increasingly clear that focusing only on these compartments of the transmission system will result in an incomplete epidemiological picture of resistance.^3^ Bacterial populations in aquatic and soil habitats are enormously diverse, and have crucial roles in nitrogen cycling, carbon sequestration, and the stability of aquatic ecosystems.^4^ These bacteria also act as reservoirs of naturally occurring bacterial resistance, the burden of which is exacerbated by flows of resistance elements and other chemicals (such as heavy metals) from livestock and human waste, which can coselect for drug resistance.^5^ Resulting changes to microbial diversity could lead to damaging effects on terrestrial and aquatic ecosystems, such as nitrification and mobilisation of heavy metals.^6^, ^7^

**Research in context**

**Evidence before this study**

We searched PubMed for the terms “wildlife”, “antimicrobial resistance”, and “urban”, with no date limits set and language limited to English. Earlier studies described antimicrobial resistance in select species of urban wildlife, and a single study compared differences in prevalence between wildlife and livestock (cattle, on UK dairy farms). No published studies examined the presence of antimicrobial resistance across diverse urban wildlife taxa inhabiting the same urban environment, and no studies compared antimicrobial resistance in sympatric wildlife, livestock, and human populations.

**Added value of this study**

Ecological and epidemiological approaches were applied to provide, to our knowledge, the first epidemiologically structured comparative analysis of phenotypic antimicrobial resistance characterisation in sympatric wildlife, livestock, humans, and the environment in an urban setting, and the most comprehensive analysis of urban wildlife-borne antimicrobial-resistant phenotypes so far. Because this study was done on a city-wide scale, it allowed us to evaluate carriage of clinically relevant antimicrobial resistance in urban wildlife across Nairobi, and relate this to antimicrobial resistance in the broader urban epidemiological system. We present several important findings, showing that, although urban wildlife carry high burdens of clinically relevant antimicrobial resistance, phenotypic diversity is lower than in humans, livestock, or the external environment. Wildlife that associate closely with livestock, humans, and both livestock and human waste are exposed to higher levels of antimicrobial-resistant phenotypes than wildlife that do not associate as closely with livestock and human waste, and could thus act as conduits for dissemination to the wider environment. Our findings emphasise the importance of understanding ecological flows of antimicrobial resistance within complex urban systems, to inform strategies aimed at limiting human exposure to multidrug-resistant bacteria.

**Implications of all the available evidence**

The results of this study and previous studies suggest that through anthropogenic exposure, wildlife have a taxa-specific role in the acquisition and dissemination of clinically relevant antimicrobial resistance across urban landscapes, and have the potential to disseminate antimicrobial resistance from urban areas to broader ecosystems. Similarly scaled future studies done in a variety of urban settings would permit examination of context-specific differences in wildlife antimicrobial resistance carriage and exposure. More broadly, contamination of urban environments with antimicrobial resistance is a serious issue, and future studies should focus on identifying antimicrobial resistant flow through urban ecological systems, and relating this to coresistance and crossresistance to other environmental pollutants (such as heavy metals). Such evidence could be used to develop cost-effective surveillance for urban ecological systems, and to inform interventions that are aimed at limiting environmental contamination with pollutants of public health significance. Ultimately, this work forms part of a broader strategy to understand the epidemiology of antimicrobial resistance across developing urban landscapes.

Wildlife exist across multiple trophic levels, and are therefore well placed to accumulate and disperse resistance determinants within ecosystems. Ecological traits, such as habitat, feeding preferences, and ranging behaviour could determine the exposure of wildlife species to antimicrobial resistance, and how widely it is dispersed in the environment.^8^, ^9^ The presence of diverse bacterial resistance profiles in wildlife inhabiting pristine environments also shows the complexity of naturally occurring antimicrobial-resistant communities in the gut of free-ranging vertebrates, for which environmental acquisition probably has an important role.^10^, ^11^ As land-use changes reduce the availability of natural habitats, wildlife species are forced to seek alternative sources of food and shelter, bringing them into closer association with humans, livestock, and their waste, and increasing the potential for transfer of antimicrobial resistance between them.^3^, ^12^

In lower-middle-income countries, urban environments act as hotspots for interactions between humans, animals, and their shared environment. The focus of this study is on the informal keeping of livestock by households in Nairobi, Kenya, as a potentially high-risk urban interface for antimicrobial-resistant transmission between wildlife, humans, livestock, and the environment. Livestock are frequently kept within household perimeters in low-income country urban centres, where differing levels of waste management could cause variation in environmental dispersal of determinants of, and exposure of wildlife to, antimicrobial resistance.^12^ Being ubiquitous in vertebrates and the environment, *Escherichia coli* is frequently targeted in studies of antimicrobial resistance, and is an ideal sentinel bacteria for the study of the dispersal of antimicrobial resistance across diverse vertebrate host species and the environment.^8^

Using *E coli* antimicrobial-resistance phenotypes collected from households across Nairobi, we explored the role of urban wildlife in the epidemiology of antimicrobial resistance. In considering antimicrobial resistance as defined by clinically significant human treatment breakpoints and to antibiotics of importance in human medicine, the true clinical relevance of antimicrobial resistance in urban wildlife is examined.^13^ Wildlife, which are not treated with antibiotics, might be a net recipient (or sink) of antimicrobial resistance in urban environments, while acting as an effective conduit of antimicrobial resistance between other parts of the system. These hypotheses are tested by using statistical models to compare the carriage of clinically relevant antimicrobial resistance between epidemiological compartments (ie, wildlife, humans, livestock, and the environment). To further understand the determinants of exposure of wildlife to antimicrobial resistance, variation in host taxon and functional ecology (eg, foraging traits) are related to carriage of multidrug-resistant *E coli*, and antibiogram length in wildlife across the city. At a finer scale, epidemiological models are used to investigate risk factors for exchange of antimicrobial resistance between sympatric wildlife, humans, and livestock, thus shedding light on pathways of antimicrobial resistance transfer at household interfaces.

## Methods

### Study design

Faecal samples (n=2081) from 75 wildlife species (birds and mammals [n=794], appendix), 13 livestock species (n=677), humans (n=333), and samples from the external environmental (n=277) were collected from households across Nairobi that were participating in the UrbanZoo 99-household project between Sept 6, 2015, and Sept 28, 2016.^14^ An additional 24 faecal samples were collected from birds and rodents in abattoirs across the city. Our study design is explained in detail in the appendix; briefly, Nairobi was split into administrative units, and 33 were chosen on the basis of a socioeconomic stratification. Three households were randomly selected in each sublocation to obtain two livestock-keeping and one non-livestock-keeping household (a total of 99 households), with the aim of maximising the spatial distribution and diversity of livestock-keeping practices captured within the sampling frame. Wildlife samples were also obtained from an additional household, where the occupants declined to submit human samples or questionnaire data. As such, 100 households were included in analyses in which isolates from wildlife were considered alone. Households in each sublocation had to meet strict inclusion criteria of keeping small ruminants or poultry, large ruminants or pigs, or no livestock within the household perimeter. Abattoirs in Nairobi were selected and sampled in a separate value chain study done as part of the wider UrbanZoo project.^15^ Wildlife samples were obtained by a range of taxon-specific trapping methods, which are described in the appendix, along with protocols for collection of human, livestock, and environmental samples. Questionnaires detailing household composition and socioeconomic data, and livestock ownership and management, were administered at each household (appendix). Household occupants who provided samples and answered questionnaires provided written consent.

The collection of data adhered to the legal requirements of the country in which the research was conducted. Wildlife were trapped under approval of an International Livestock Research Institute (ILRI) Institutional Animal Care and Use Protocol (IACUC; 2015.12), and permits obtained from the National Museums of Kenya and Kenya Wildlife Service. Livestock samples were obtained under approval of ILRI IACUC (2015.18). Human samples and questionnaire data were collected under approval of ILRI Institutional Research Ethics Committee approval (2015-09).

### Microbiological testing

All rectal swabs and fresh faecal samples were placed in Amies transport media, and transported on ice to one of two laboratories (Kenya Medical Research Institute or University of Nairobi [UoN]). Boot socks (on which surface material from livestock pens and the external environment were collected) and modified Moore swabs were transported in saline-filled polythene bags, and water samples were transported in conical tubes, all on ice. Samples were enriched in buffered peptone water for 24 h, and then plated onto eosin methylene blue agar (EMBA) and incubated for 24 h at 37°C. Subsequently, five colonies were selected and subcultured on EMBA, before being further subcultured on Müller-Hinton agar and stored at −20°C in cryovials. A single colony was picked at random from the plate for each original sample (ie, an isolate) and biochemical tests (triple sugar iron agar, Simmon's citrate agar, and motility-indole-lysine media) were used for presumptive identification of *E coli*. A single colony was picked from each avian or bat pooled faecal sample.

All isolates were revived and inoculated onto Müller-Hinton plates before antimicrobial susceptibility testing. Isolates were tested for susceptibility to ampicillin (10 μg), amoxicillin–clavulanic acid (30 μg), cefepime (30 μg), cefotaxime (30 μg), ceftazidime (30 μg), chloramphenicol (30 μg), nalidixic acid (30 μg), ciprofloxacin (5 μg), gentamicin (10 μg), streptomycin (25 μg), sulfamethoxazole (30 μg), tetracycline (30 μg), and trimethoprim (2·5 μg) using the disc diffusion method according to the Clinical and Laboratory Standards Institute guidelines.^13^ Antibiotics included those frequently used in both veterinary and human medicine in Kenya.^16^ Clinical and Laboratory Standards Institute guidelines were also used to determine human breakpoints for classifying isolates as sensitive, intermediate, or resistant to the drug.^13^ Following previous studies, intermediate strains were deemed to be moving towards resistance, and thus considered resistant on an evolutionary basis.^17^, ^18^ All protocols were standardised between laboratories, and between-laboratory quality control was done at regular intervals. Multidrug-resistant *E coli* was defined as “non-susceptibility to at least one agent in three or more antimicrobial classes”^17^ (appendix). Wildlife isolates were also assessed for high levels of multidrug resistance (non-susceptibility to at least seven antimicrobial classes tested) and resistance to all antibiotics tested for in this study. An antibiogram was defined as the combination of antibiotics to which an isolate was resistant, and thus antibiogram length was defined as the number of antibiotics to which an isolate was phenotypically resistant.

### Statistical analysis

All statistical analyses were done using R, version 3.3.2. Spatial structure in the dataset was represented using distance-based Moran's eigenvector maps—a powerful multivariate approach to model spatial structure in a response variable, which can be partitioned at broad, medium, and fine spatial scales.^19^, ^20^ Further details of how we dealt with missing data, data exploration, and statistical models (distributions, choice of fixed and random effects, implementation, and model selection procedures) are given in the appendix.

To test the hypothesis that urban wildlife are a net source or sink of antimicrobial resistance in Nairobi when compared with humans, livestock, and the environment, epidemiological and ecological statistical modelling approaches were applied. Prevalence of resistance to 13 antibiotics was compared between all four epidemiological compartments (ie, wildlife, human, livestock, environment) in a Bayesian analysis framework, using Markov Chain Monte Carlo methods.^21^, ^22^ Generalised linear mixed effects models (GLMMs) with binomial (log-link function) and Poisson distributions were used to test whether multidrug-resistant *E coli* carriage and antibiogram length differed between compartments, and how this varied spatially across the city. To assess how antibiogram diversity was distributed across compartments, antibiogram diversity was compared using four ecological measures of diversity related to Rényi's measures of generalised entropy.^23^ Methods adapted from community ecology were used to extend the comparison of phenotypic diversity between compartments by estimating the number of undetected antibiograms. Chao2, ICE, and Jack-knife incidence-based statistical methods were used to estimate the minimum total antibiogram richness in each compartment from the data, by looking at frequencies of phenotype occurrence in collections of individuals. To consider the implications for surveillance, methods from Chao and colleagues^24^ were followed to estimate the sampling effort required to detect a given proportion of the total antibiograms estimated for each compartment. Our approach is described in full in the appendix.

A Bayesian analysis framework, as described earlier, was used to estimate and compare prevalence of resistance to 13 antibiotics between wildlife taxa. Ecological traits considered potentially important factors for exposure of wildlife to antimicrobial resistance were modelled against multidrug-resistant *E coli* carriage and antibiogram length in wildlife in binomial and Poisson GLMMs, respectively. Separate binomial GLMMs were developed to investigate fine-scale household-level risk factors for the likelihood of multidrug-resistant *E coli* carriage in select urban wildlife with synanthropic traits (ie, rodents and seed-eating birds). Risk factors were sourced from a set of anthropogenic and ecological covariates capturing antimicrobial-resistant *E coli* carriage in humans and livestock, livestock-keeping practices, land use within households, and ranging behaviour of wildlife. All anthropogenic and ecological variables were derived from metadata collected within households, and published sources (appendix). The laboratory in which samples were tested was included as a confounding factor in these models.

### Role of the funding source

The funders of the study had no role in the study design, data collection, data analysis, or interpretation. JMH and EMF always had full access to the data in the study, and had final responsibility for the decision to submit for publication.

## Results

Samples were collected from 547 individual birds, nine avian populations (31 pooled samples across nine populations), 167 rodents, 44 individual bats, five bat populations (20 pooled samples across nine populations), five carnivores, and four primates across 100 households, as well as from 11 abattoirs in Nairobi, between Sept 6, 2015, and Sept 28, 2016. Antimicrobial susceptibility tests were done on a single *E coli* isolate cultured from 282 (52%) of 547 birds, 20 (65%) of 31 avian populations, 155 (93%) of 167 rodents, 22 (50%) of 44 bats, six (22%) of 27 bat populations, three (60%) of five carnivores, and four (100%) of four primates. Because of low sample numbers, primates and carnivores were not included in further statistical analysis, and each pooled population sample was considered as coming from an individual bird or bat for the purposes of all further analysis. *E coli* was isolated from, and antimicrobial susceptibility tests done on, 638 livestock, 321 human, and 256 environmental samples. 252 (52%) of 485 samples from wildlife sampled in Nairobi carried multidrug-resistant *E coli*; eight (2%) of 485 wildlife isolates (all originating from birds) carried *E coli* resistant to agents belonging to at least seven of the antimicrobial classes tested; and *E coli* isolated from a single avian sample was resistant to all antimicrobials tested.

**Figure 1:**
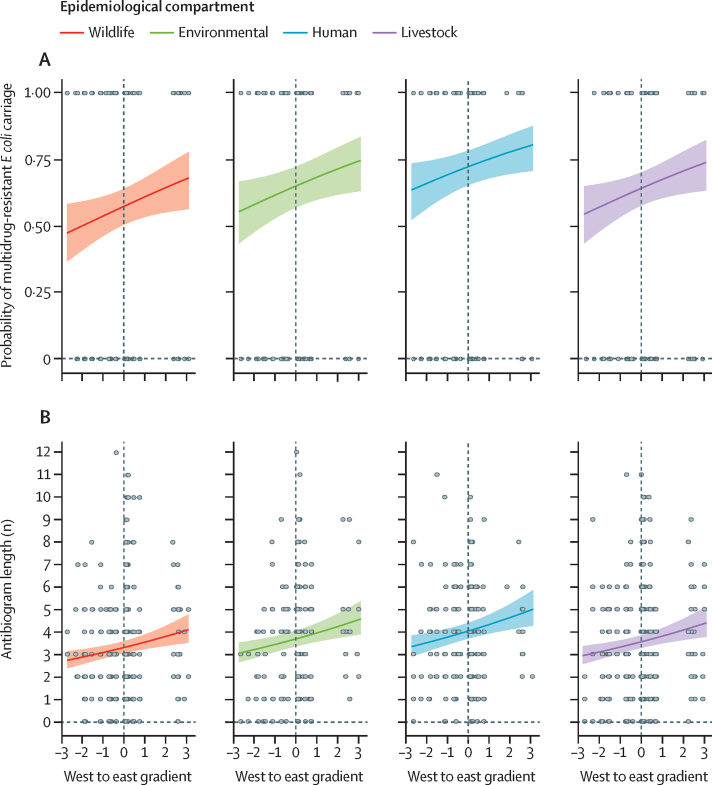
Variation in probability of multidrug resistant *Escherichia coli* carriage (A) and antibiogram length (B) in different epidemiological compartments along a west to east gradient across Nairobi Coloured shading represent 95% CI.

Prevalence of antimicrobial-resistant *E coli* in wildlife was significantly lower than at least one epidemiological compartment for six of the antibiotics tested (ampicillin [human], cefepime [livestock], cefotaxime [livestock], streptomycin [human], tetracycline [human, livestock, environment], and trimethoprim [human]), and not significantly higher than other compartments for any of the 13 antibiotics tested (appendix). Wildlife were less likely to carry multidrug-resistant *E coli* than humans and livestock (β=0·662, 95% CI 0·36 to 0·97, p<0·0001; β=0·284, 95% CI 0·03 to 0·53, p=0·026), and had shorter antibiogram lengths than all other compartments (marginal R^2^ 0·028; table 1). Five distance-based Moran's eigenvector maps were associated with multidrug-resistant *E coli* carriage and antibiogram length of isolates, and were thus included as covariates in the GLMMs. Both models showed broad-scale spatial relationships for antimicrobial resistance carriage across the city; the probability of multidrug-resistant *E coli* carriage in all epidemiological compartments increased along a west to east gradient (MEM1; β=0·15, 95% CI 0·02 to 0·28, p=0·026; marginal R^2^ 0·028; figure 1), whereas antibiogram lengths decreased from eastern to western Nairobi (MEM1) and increased from northern to southern Nairobi (MEM2; β=0·07, 95% CI 0·03 to 0·11, p=0·00093; β=–0·04, 95% CI −0·08 to 0, p=0·041; marginal R^2^ 0·028, 0·043; figure 1).

**Table 1:** Estimated regression parameters, SEs, Z scores, and p values for generalised linear mixed models

	**Estimate**	**SE**	**Z score**	**p value**
**Model: MDR carriage in all isolates**				
Intercept	0·030	0·102	0·296	0·77
Environment	0·322	0·165	1·954	0·051
Human	0·662	0·155	4·273	<0·0001
Livestock	0·284	0·128	2·222	0·026
MEM1	0·148	0·067	2·225	0·026
MEM2	−0·118	0·066	−1·781	0·075
MEM5	−0·130	0·063	−2·072	0·038
**Model: antibiogram length of all isolates**				
Intercept	1·095	0·031	35·8	<0·0001
Environment	0·107	0·044	2·42	0·015
Human	0·199	0·040	4·94	<0·0001
Livestock	0·070	0·036	1·96	0·049
MEM1	0·070	0·021	3·31	0·00093
MEM2	−0·042	0·020	−2·04	0·041
MEM5	−0·049	0·019	−2·60	0·0095

SE=standard error. MDR=multidrug resistance. MEM1, MEM2, and MEM5 indicate the spatial scales across which variation in MDR carriage or antibiogram length occurs.

**Figure 2:**
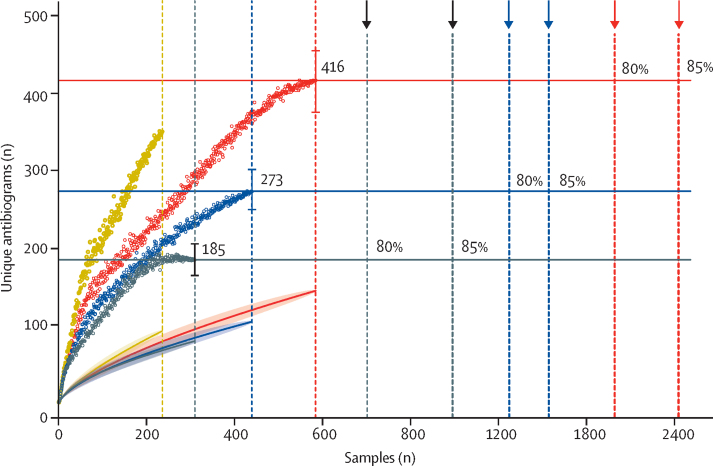
Asymptotic antibiogram richness estimates for each epidemiological compartment Dotted curves indicate Chao2 estimators at every sample point (95% CIs indicated by bars at asymptote). Horizontal lines indicate asymptotic estimate of antibiogram richness for each compartment. Shaded curves indicate species accumulation curves (line represents model fitted values, shaded areas represent 95% CIs). Vertical dotted lines indicate number of samples collected from each compartment. Vertical dashed lines indicate sampling effort required to detect 80% and 85% of the asymptotic estimate for antibiogram richness in each compartment.

Population-diversity measures of resistance indicated that wildlife had less diverse antibiograms than other compartments. *E coli* isolated from wildlife had a lower expected antibiogram diversity than all other compartments as measured by three of the four *D*_α_ diversity indices calculated (Shannon entropy, Simpson diversity, and Berger-Parker; appendix). When compared across all compartments, the range of median α values was significantly lower in wildlife than all other compartments (wildlife:environmental p=0·0079; wildlife:livestock p=0·002; wildlife:human p=0·00021). Asymptotic estimates of minimum total antibiogram richness in wildlife were 273 (95% CI 245–300) unique antibiograms, most of which could be detected if an additional 8848 samples were collected (figure 2; appendix). This richness estimate is lower than estimates for the environment (350, 95% CI 305–395) and livestock (416, 378–454), but higher than the estimate for humans (185, 165–205). Unlike the human compartment, where an asymptote was reached at 270 samples, wildlife and livestock estimates were only beginning to reach an asymptote at the sampling extremes achieved in this study. None of the statistical estimators reached an asymptote for environment, suggesting that the rate of discovery of new antibiograms in this compartment was still high, and minimum richness estimates could therefore be considerably higher than 350.

When split into taxonomic groups, prevalence of *E coli* isolates susceptible to all antibiotics tested was 45 (9%) of 485 samples across all wildlife, 26 (9%) of 282 birds, two (10%) of 20 avian populations, 13 (8%) of 155 rodents, and four (14%) of 28 bats. Bayesian models showed that prevalence of resistance to streptomycin, tetracycline and trimethoprim varied significantly between wildlife when stratified by taxonomic or functional groups (appendix). Birds belonging to the orders Pelecaniformes and Ciconiiformes were more likely to carry *E coli* resistant to ceftazidime (odds ratio 7·9, 95% CI 1·7–28·5; p=0·0033), and had significantly longer antibiograms than other species of wildlife (p=0·04).

**Figure 3:**
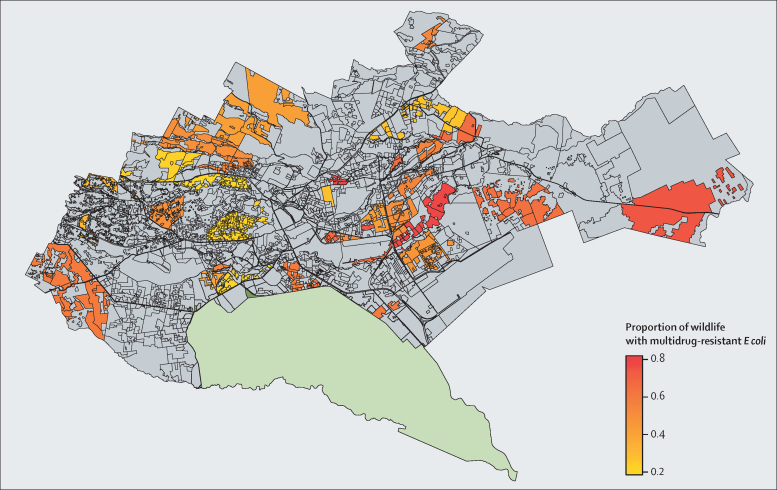
Proportion of wildlife carrying multidrug-resistant *Escherichia* co*li*, stratified by the sublocation in Nairobi in which they were sampled

Multidrug-resistant *E coli* carriage varied by taxonomic functional groups, and along an east to west gradient across Nairobi (marginal R^2^ 0·08; figure 3). Frugivorous bats and seed-eating, omnivorous, and scavenging birds were significantly more likely to carry multidrug-resistant *E coli* than frugivorous birds, and the probability of carrying multidrug-resistant *E coli* increased significantly from west to east Nairobi (appendix). *E coli* antimicrobial resistant antibiograms were longer in birds than rodents (β=–0·16, 95% CI −0·29 to −0·03, p=0·016), and antibiogram length showed spatial correlation across multiple scales of the city (broad-scale [east to west; MEM1], medium-scale [MEM8, 10, 19], and fine-scale [MEM25, MEM27] resolutions; marginal R^2^ 0·13; appendix). Wildlife-borne *E coli* processed at UoN laboratories had significantly longer antibiograms. The effects of laboratory were only present in a single model, and all reasonable efforts were taken to ensure that protocols were standardised between laboratories; specifically, a postdoctoral researcher was responsible for ensuring that these standards were maintained throughout the project. As such, although this variation could have arisen through operator bias, it is likely to have had a limited effect, if any, on our results.

**Figure 4:**
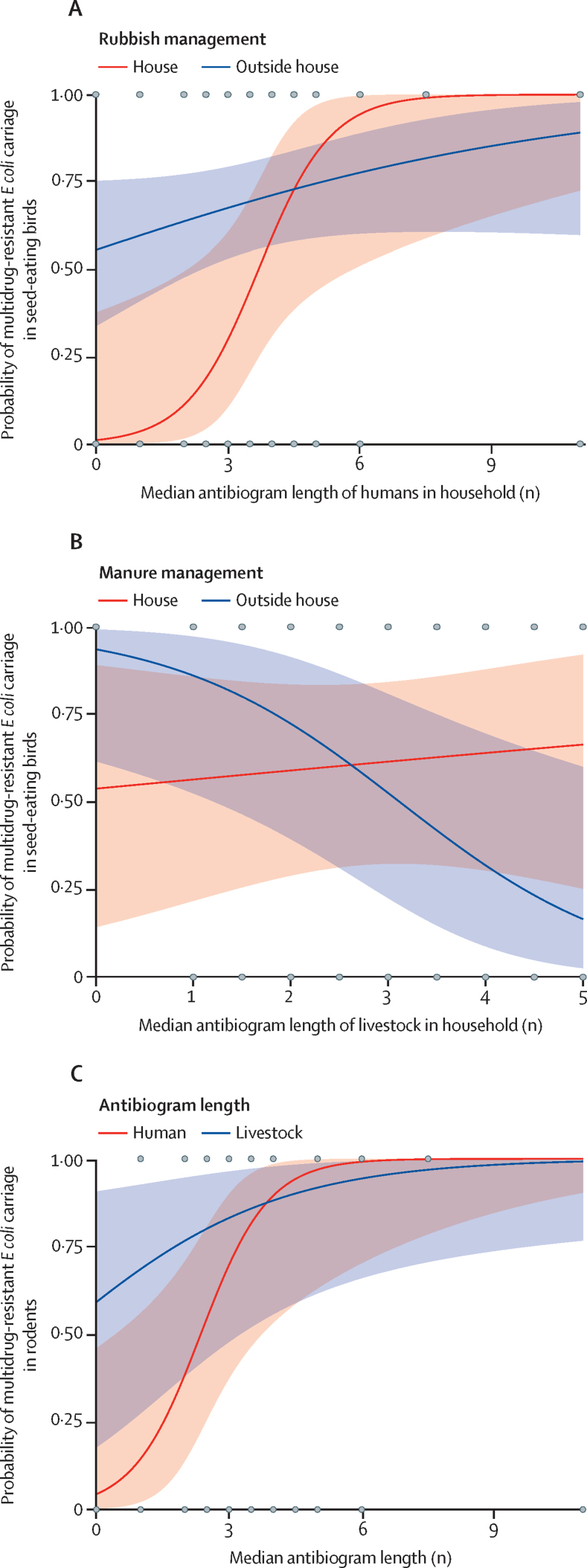
Fit of the binomial generalised linear mixed effects models relating multidrug-resistant *Escherichia coli* and carriage in birds and rodents to household-level anthropogenic and ecological covariates (A) The effects of different rubbish management on the relationship between the probability of multidrug-resistant *E coli* carriage in seed-eating birds and antibiogram length in humans. (B) The effects of different manure management on the relationship between the probability of multidrug-resistant *E coli* carriage in seed-eating birds and antibiogram length in livestock. (C) Human and livestock antibiogram lengths in a household and the probability of multidrug-resistant *E coli* carriage in rodents. All other covariates in the models are kept constant. Shading indicates 95% CIs, and grey points are individual data points.

Seed-eating birds and rodents, which are ubiquitous in households across Nairobi and frequently display anthropophilic (human-associated) feeding behaviour, were used as the basis of efforts to understand antimicrobial resistance overlap within households. In any given household, the likelihood of carriage of multidrug resistance in seed-eating birds was best described by increasing numbers of cattle in the household perimeter, and antibiogram length of the human inhabitants (β=3·41, 95% CI 1·42–5·4, p=0·00078; β=1·22, 95% CI 0·16–2·29, p=0·025; R^2^ 0·3; table 2). The relationship between human antibiogram length and avian carriage of multidrug resistance was affected by whether rubbish was kept within the household perimeter or not (β=4.76, 95% CI 0·76–8·76, p=0·02); keeping rubbish within the perimeter resulted in a stronger relationship between human antibiogram length and avian carriage of multidrug resistance (figure 4A). When manure was kept inside the household perimeter, the probability of carriage of multidrug resistance in seed-eating birds increased with longer antibiogram lengths in livestock, whereas the opposite was true when manure was disposed of externally (figure 4B). The likelihood of multidrug resistance carriage in rodents increased with increasing antibiogram length of human and livestock inhabitants in the household (β=1·31, 95% CI 0·25–2·37, p=0·015; β=0·41, 95% CI 0·03–0·79, p=0·035; R^2^ 0·42; figure 4C; table 2). Although not statistically significant within the model, keeping both rubbish and manure outside the household perimeter reduced the likelihood of rodents carrying multidrug resistance as human antibiogram length increased.

**Table 2:** Estimated regression parameters, SEs, Z scores, and p values for generalised linear mixed models

	**Estimate**	**SE**	**Z score**	**p value**
**Model: MDR carriage in seed-eating birds**				
Intercept	−5·4935	2·3398	−2·348	0·019
Total cattle	3·4136	1·0158	3·361	0·00078
Human ABG	1·2222	0·5443	2·245	0·025
Livestock ABG	0·1056	0·2893	0·365	0·72
Manure (outside house)	2·5294	1·4222	1·779	0·075
Garbage (outside house)	4·7585	2·0421	2·320	0·02
Garbage (outside house), human ABG	−1·0513	0·5332	−1·972	0·049
Manure (outside house), livestock ABG	−0·9655	0·4708	−2·051	0·04
**Model: MDR carriage in rodents**				
Intercept	−4·3039	1·7504	−2·459	0·014
Human ABG	1·3059	0·5383	2·426	0·015
Livestock ABG	0·4085	0·1942	2·104	0·035
Manure (outside house)	2·9078	1·2650	2·299	0·022
Garbage (outside house)	1·4198	1·6627	0·854	0·39
Laboratory (University of Nairobi)	−2·0261	1·1738	−1·726	0·084
Garbage (outside house), human ABG	−1·0043	0·5821	−1·725	0·085
Manure (outside house), human ABG	−0·5909	0·3290	−1·796	0·073

SE=standard error. MDR=multi-drug resistant *Escherichia coli.* ABG=antibiogram length.

## Discussion

We show that urban wildlife species are important components of the environmental pool of resistance to clinically relevant antimicrobials, and through exposure mediated by resource provisioning, could be involved in disseminating clinically relevant resistance across landscapes (appendix). Unlike most previous studies on antimicrobial resistance in wildlife, in which wild animals have been opportunistically sampled,^11^ we used an epidemiological study design to compile a large bacterial dataset for investigation burdens of antimicrobial resistance in sympatric wildlife, humans, and livestock, and their shared environment.

High numbers of *E coli* resistant to clinically relevant antibiotics were detected in urban wildlife, including resistance to the more newly developed drugs such as third-generation cephalosporins, and synthetic fluoroquinolones, which WHO considers crucial for human medicine.^25^ *E coli* that produce extended-spectrum β-lactamase enzymes, which generally confer resistance to cephalosporins, are a major concern to human and veterinary medicine worldwide, and have been frequently reported in wildlife.^26^ However, livestock and environmental compartments (both of which had higher ecological diversity of antimicrobial resistance, higher prevalence of multidrug resistance, longer antibiogram length, and with which humans have more direct contact) yield more potential as routes of human exposure to novel antimicrobial resistance genes in Nairobi. As such, our results are consistent with the hypothesis that wildlife are not a net source of antimicrobial resistance diversity in Nairobi, and probably pose little direct threat to human health in the urban areas. The estimate of total antimicrobial resistance richness in humans was considerably lower than that of all other compartments. This difference, which was robustly supported by statistical estimators, might indicate that, compared with humans, wildlife and livestock are exposed to greater antimicrobial resistance diversity through their closer interaction with the environment.

The vertebrate gastrointestinal microbiome plays a key role in the population structure for genes conferring resistance to antimicrobials, and microbiome composition is directed by an array of factors linked to host genotype, age, and diet.^27^ Although the direct effects of diet and physiological factors on selection for faecal antimicrobial resistance genes could not be assessed, our results are broadly supportive of previous studies that report that anthropophilic omnivores and carnivores have a higher risk of carrying, and potentially spreading, antimicrobial-resistant bacteria.^8^ In this study, scavenging birds and water birds had longer antibiograms than all other wildlife species. Antimicrobial resistance-carriage in high proportions of water birds is a common finding in other parts of the world,^28^ where, in the absence of natural habitats such as wetlands, these species forage on sewage treatment plants, rubbish dumps, and abattoir viscera ponds. Artificial habitats such as these are considered important routes for the dispersal of human-excreted and livestock-excreted antimicrobial resistance into the environment.^11^, ^29^

Within households, increasing likelihood of multidrug-resistant *E coli* carriage in synanthropic wildlife as phenotypic antimicrobial resistance diversity in sympatric livestock and humans also increases suggests transfer of clinically relevant antimicrobial resistance between humans and livestock, and certain wildlife species. These associations were more pronounced for seed-eating birds in the presence of manure and rubbish, indicating that human and livestock waste are conduits for the transfer of antimicrobial resistance between humans, livestock, and peridomestic birds, with the potential for dissemination of antimicrobial resistance phenotypes into the wider environment. Manure can be a reservoir for the amplification of antimicrobial resistance determinants, particularly plasmids.^30^ These results support those of other studies^31^, ^32^, ^33^ that have identified the importance of provision of urban resources in bringing wildlife into closer association with humans and livestock, offering new opportunities for disease transmission. However, although our results are suggestive of antimicrobial resistance exchange, transmission cannot be inferred from overlap of phenotypic antimicrobial resistance and, as such, genetic data are required to corroborate the existence of interfaces for antimicrobial resistance exchange, and determine the direction in which bacteria or resistance elements are being transferred. We aim to address this in forthcoming studies.^34^ More broadly, wildlife–livestock–human interfaces such as these represent a crucial point for cross-species transmission, and emergence of pathogens into new host populations.^12^ Removal of manure and rubbish (sources of anthropogenic resource provision) from households reduced the magnitude of antimicrobial resistance exposure in seed-eating birds, either through limiting wildlife–livestock or wildlife–human contact or reduced exposure of wildlife to sources of antimicrobial resistance.

Complex urban systems such as those of Nairobi are a feature of many lower-middle-income countries, and our findings are therefore broadly applicable to the urban epidemiology of antimicrobial resistance in these countries. High proportions of antimicrobial resistance and multidrug-resistant *E coli* carriage in wildlife could be indicative of environmental antibiotic contamination, and high background levels of antimicrobial resistance in Nairobi's urban environment (supported by our findings of high phenotypic diversity in environmental samples). Clinically relevant resistance genes were thought to be rare in soils in the preantibiotic era and, as such, it is to be expected that the urban environmental resistome (the collection of resistance determinants present in pathogenic and non-pathogenic bacteria in the soil) in rapidly developing cities such as Nairobi is heavily influenced by human activity.^35^ However, interactions between naturally occurring and anthropogenic-derived antimicrobial resistance determinants in bacteria occurring in the broader urban environment, outside urban reanimation units, are poorly understood. The geospatial, temporal, chemical, and biological complexities of urban systems make this a particularly challenging topic of study.

If wildlife exposure to antimicrobial resistance is largely determined by habitat use, targeted surveillance of wildlife that frequent high-risk urban environmental interfaces (where the accumulation of antibiotic residues or other coselecting agents, such as heavy metals, might force the accelerated evolution and fixing of resistance determinants) could be an efficient way to detect clinically important determinants of resistance. To explore the practicality of surveillance in wildlife, the sampling effort required to detect different fractions of the total estimated antimicrobial resistance richness was calculated (figure 2; appendix). To detect all 273 predicted antibiograms in the wildlife species sampled would require an extra 8848 samples, an impractical and expensive task. However, detecting 85% of the total diversity would require a disproportionately lower sampling effort of 1572 samples. Assuming that the diversity of antibiograms in selected wildlife is lower than the total diversity represented by all taxonomic classes of wildlife included in this study, the required sampling effort to achieve an acceptable likelihood of detecting new antibiograms in these species would be much lower. Extending this approach to livestock and humans reveals similar outcomes for surveillance of antibiograms in these compartments (figure 2), suggesting that practical and economically viable surveillance for antimicrobial resistance of public health concern in urban wildlife, livestock, and humans could be achieved through targeted longitudinal surveillance, designed to capture a high proportion of diversity at regular intervals.

Urban ecosystems with high levels of background environmental antimicrobial resistance could act as pools of antimicrobial resistance dissemination to peripheral ecosystems, where the flow of water, and movement of humans, livestock, and wildlife act as vectors for dispersal.^29^ Although little is known about how resistance genes are carried and shed by wildlife species,^11^ previous studies reporting extended-spectrum β-lactamase *E coli* carriage in migratory wild birds, and carriage of bacteria with resistance to more antibiotics than non-migratory wild birds,^36^ indicate that wildlife could have an important role in disseminating clinically relevant antimicrobial resistance across landscapes. Our finding of higher levels of antimicrobial resistance carriage in birds (particularly scavenging birds with large home ranges) than other species suggest that these species could disseminate antimicrobial resistance determinants to neighbouring ecosystems—Nairobi is surrounded by a complex patchwork of high-density human populations, natural areas, forest, and rangelands. Mapping the distribution of multidrug-resistant *E coli* in wildlife by sublocation shows high levels of multidrug resistance carriage extending to peripheral areas of Nairobi, which border rich Savannah ecosystems to the south and east of the city (figure 3). Nairobi National Park, which borders the city to the south, is home to a high density of migratory wildlife species that could disperse antimicrobial resistance genes to more distant areas.^37^ Our models for antimicrobial resistance carriage showed a clear east to west gradient, indicating that wildlife antimicrobial resistance diversity is higher in the east of the city, which corresponds to the extreme environmental, ecological, and social gradients that split Nairobi in east to west. Such extreme differentiation within a single city shows the highly complex ecosystem within which the urban epidemiology of antimicrobial resistance is set.

This study has several limitations. Although the prevalence of resistance to individual antibiotics and multidrug-resistant *E coli* carriage in wildlife was high, without comparable datasets from other urban or rural settings it is difficult to say how unique these results are to Nairobi. Studies done in a variety of urban settings, and considering high-risk sites of environmental antimicrobial resistance contamination beyond the household scale, would permit examination of context-specific differences in wildlife antimicrobial resistance carriage and exposure. Because of the effort required to sample wildlife of different species our sample size was small for cryptic taxonomic and functional groups (eg, bats, scavengers, and frugivores or nectarivores). In addition, by only culturing a single isolate from each host, the within-host diversity of antimicrobial-resistant *E coli* was not considered. We made this decision as a necessary, cost-based trade-off between microbiological resolution and sample size. However, the effects of restricted sample size would only act to increase type II error in our results (ie, conservative statistical inference, or missed signal in the data), and are thus unlikely to affect the validity of our findings. More broadly, our focus on mammalian and avian urban wildlife neglects the role of reptiles, aquatic organisms, and invertebrates. Studies investigating the effects of antimicrobial resistance on invertebrates, and their role in carriage and dispersal of resistance elements are warranted given the indispensable role invertebrates play as pollinators, biocontrol agents, and in the degradation and recycling of organic matter in soils.^38^, ^39^

To conclude, carriage of clinically relevant antimicrobial-resistant phenotypes in urban wildlife collected from households in Nairobi is predicted by feeding ecology, and interaction with humans, livestock, and both human and livestock waste. Even if clinical use is the main driver for the emergence of antimicrobial resistance in humans, environmental compartments such as wildlife can accumulate clinical residues, be reservoirs for novel antimicrobial resistance genes, and have the potential to disseminate resistance determinants across urban landscapes. This potential means that there is a pressing need to consider the ecosystem-wide epidemiology of antimicrobial resistance in urban environments. As Robinson and colleagues^40^ speculate, poorly enforced environmental legislation and unregulated antibiotic use might render these factors more pronounced in developing countries. Further studies and targeted surveillance, which take a similarly broad approach to epidemiological compartments, will be required to consider how the genetic determinants of resistance are passed between compartments and disseminated into the wider environment.

For **data** see http://dx.doi.org/10.17638/datacat.liverpool.ac.uk/729

For the **CGIAR Fund Donors** see https://www.cgiar.org/funders/

## Data sharing

Data (antimicrobial resistance sensitivity testing datasets, and accompanying metadata) are available via an open access repository held by the University of Liverpool.
