# Correction to *Lancet Planet Health* 2019; 3: e259–69

**DOI:** 10.1016/S2542-5196(19)30105-6

**Published:** 2019-07

*Hassell JM, Ward MJ, Muloi D, et al. Clinically relevant antimicrobial resistance at the wildlife–livestock–human interface in Nairobi: an epidemiological study.* Lancet Planet Health *2019; **4**: e259–69*—In figure 2 of this Article, the key was omitted. This correction has been made as of July 17, 2019.

