# Supplementary material for: Clinically relevant antimicrobial resistance at the wildlife–livestock–human interface in Nairobi: an epidemiological study
Source: Lancet Planet Health. 2019 Jun;3(6):e259–69. doi: 10.1016/S2542-5196(19)30083-X (PMC6630895; doi:10.1016/S2542-5196(19)30083-X)
Supplement: Supplementary appendix [file mmc1.pdf]

THE LANCET
Planetary Health

Supplementary appendix

This appendix formed part of the original submission and has been peer reviewed. We post it as supplied by the authors.

Supplement to: Hassell J M, Ward M J, Muloi D, et al. Clinically relevant antimicrobial resistance at the wildlife–livestock–human interface in Nairobi: an epidemiological study. *Lancet Planet Health* 2019: **3:** e259–69.

Lancet Supplementary Material, to accompany the manuscript "Epidemiology of Clinically Relevant Antimicrobial Resistance at the Wildlife-Livestock-Human Interface in Nairobi" by Hassell et al.

Supplementary Tables and Figures

Bat	Rodent	Bird	Carnivore	Primate
Eidolon spp.	Mus spp.	Abyssinian Citril	Cape clawless otter	Sykes Monkey
Epomorphus spp.	Rattus spp.	African Firefinch	White-tailed mongoose	Vervet monkey
Minioptera spp.	Cricetomys spp.	African Paradise-flycatcher		
Neoromicia spp.	Grammomys spp.	African Pied Wagtail		
Pipistrellus spp.	Mastomys spp.	African Reed Warbler		
Rousettus spp.	Crocidurinae	African Sacred Ibis		
Scotophilus spp.		Amethyst Sunbird		
		Baglafecht Weaver		
		Barn Swallow		
		Black-headed Weaver		
		Bronze Munia		
		Bronze Sunbird		
		Cape Robin-chat		
		Cattle Egret		
		Collared Sunbird		
		Common Bulbul		
		Common Fiscal		
		Egyptian Goose		
		Fischer's Lovebird		
		Fischer's Sparrow-lark		
		Green-headed Sunbird		
		Hadada Ibis		
		House Sparrow		
		Hunter's Cisticola		
		Kenya Rufous-sparrow		
		Laughing Dove		
		Lesser Swamp-warbler		
		Marabou Stork		
		Montane White-eye		
		Northern Double-collared Sunbird		
		Northern Grey-headed Sparrow		
		Olive Thrush		
		Parrot-billed Sparrow		
		Pied Crow		
		Pin-tailed Whydah		
		Red-billed Firefinch		
		Red-cheeked Cordonbleu		
		Red-eyed Dove		
		Red-faced Cisticola		
		Rock Martin		
		Rueppell's Robin-chat		
		Scarlet-chested Sunbird		
		Singing Cisticola		
		Speckled Mousebird		
		Streaky Seedeater		
		Variable Sunbird		
		Village Indigobird		
		Village Weaver		
		White-browed Robin-chat		
		White-browed Sparrow-weaver		
		White-eyed Slaty Flycatcher		
		White-winged Widowbird		
		Winding Cisticola		
		Yellow Bishop		
		Yellow Wagtail		
		Yellow-breasted Apalis		
		Yellow-rumped Seedeater		
		Yellow-whiskered Greenbul		

Table S1. List of wildlife species from which samples were collected for this study, identified to genus for bats and rodents, and common name/species for birds, carnivores and primates.

Antibiotic Class	Antibiotic Drug
Aminoglycoside	Gentamicin
	Streptomycin
b-lactam	Amoxicillin-clavulonic Acid
Cephalosporin	Cefepime
	Cefotaxime
	Ceftazidime
Chloramphenicol	Chloramphenicol
Folic Acid	Trimethoprim
Penecillin	Ampicillin
Quinolone/Fuoroquinolone	Cirpofloxacin
	Nalidixic Acid
Sulfonamide	Sulfamethoxazole
Tetracycline	Tetracycline

Table S2. Antibiotic drugs and their classes, considered in this study.

Table S3. Ecological and anthropogenic covariates considered in this study. See supplementary webappendix references for literature cited in this table.

	Covariate	Format	Project source	Source	Notes
**Ecological**	Taxonomic group	Categorical (Avian popultaion, Avian, Bat, Rodent)	Metadata		
	Feeding niche	Categorical (Fruit/Nectar, Omnivore, Invertebrate, Seeds, Scavenger)			
	Epidemiological compartment	Categorical (Wildlife, Environment, Livestock, Humans)	Metadata	[18]	
	Proportion of time spent foraging on ground	Continuous (Proportion)		[18]	
	Home range	Continuous (Hectares) (Log[10])	Metadata (body weight)	[8, 20-22]	Not calculated for bats
	Association of species with water	Binary (Yes/No)		[19]	
	Proportion of household habitat consisting of cropland	Continuous (Proportion)	Ecological mapping		
	GPS coordinates for each sample		Metadata		
**Anthropogenic**	Presence of livestock in household	Binary (Yes/No)	Household metadata		
	Total humans in household	Continuous (Log[10])	Household metadata		
	Number of individual livestock species in household (cattle, goats, sheep, pigs, indigenous poultry, exotic poultry, lagomorphs, waterfowl, pigeons)	Continuous (Log[10])	Household metadata		
	Median profile length in humans in household	Continuous	AST data		
	Median profile length in livestock in household	Continuous	AST data		
	Median profile length in household environmental samples	Continuous	AST data		
	Household garbage (rubbish) management	Categorical (Inside house, Outside house)	Household metadata		
	Household manure management	Categorical (Inside house, Outside house)	Household metadata		
	Position of household on wealth index	Continuous	Household metadata		
	Week of the year in which samples were collected	Continuous	Household metadata		
	Laboratory samples were tested in	Categorical (Kemri/UoN)	AST data		

Table S4. Bayesian prevalence estimates (and 95% CI) for antimicrobial resistance to different drugs in wildlife (stratified by taxonomic group, and functional group), and across wildlife, livestock, human and environmental compartments. LCI and UCI are lower and upper confidence intervals respectively. Prevalences coloured blue and red indicate statistically significant differences in prevalence between groups.

		Compartment				Wildlife Taxon				Feeding Niche				
		Wildlife	Human	Livestock	Environment	Avian	Rodent	Bat	Avian population	Fruit/Nectar	Invertebrate	Omnivore	Seed	Scavenger
**Antimicrobial Phenotype**	**Amoxicillin C**													
	Prevalence	**6.42**	**9.04**	**5.35**	**10.94**	**8.14**	**3.24**	**0.00**	**15.21**	**0.00**	**7.76**	**2.60**	**8.65**	**13.22**
	LCI (95% CI)	*4.35*	*6.13*	*3.69*	*7.39*	*5.32*	*1.11*	*NA*	*3.44*	*NA*	*3.06*	*0.65*	*4.95*	*1.79*
	UCI (95% CI)	*8.85*	*12.53*	*7.15*	*15.08*	*11.60*	*6.58*	*NA*	*33.48*	*NA*	*14.62*	*5.52*	*13.29*	*33.54*
	**Ampicillin**													
	Prevalence	**33.80**	**46.45**	**33.87**	**35.95**	**34.39**	**31.06**	**35.62**	**45.02**	**25.97**	**31.73**	**33.11**	**38.03**	**53.32**
	LCI (95% CI)	*29.72*	*41.44*	*30.29*	*30.22*	*29.05*	*24.11*	*19.75*	*25.02*	*11.33*	*21.71*	*25.94*	*31.02*	*28.69*
	UCI (95% CI)	*38.14*	*51.68*	*37.54*	*42.07*	*40.12*	*38.47*	*52.88*	*66.20*	*43.99*	*42.60*	*41.25*	*45.25*	*77.29*
	**Chloramphenicol**													
	Prevalence	**5.74**	**7.80**	**4.83**	**5.10**	**8.15**	**0.64**	**6.94**	**9.89**	**10.91**	**7.63**	**1.91**	**8.00**	**13.49**
	LCI (95% CI)	*3.88*	*5.12*	*3.30*	*2.73*	*5.32*	*0.02*	*0.95*	*1.34*	*2.29*	*2.99*	*0.40*	*4.35*	*1.63*
	UCI (95% CI)	*7.87*	*10.90*	*6.63*	*8.17*	*11.49*	*2.41*	*18.52*	*25.93*	*24.27*	*14.46*	*4.51*	*12.49*	*33.60*
	**Ceftazidime**													
	**Prevalence**	**5.78**	**7.80**	**4.85**	**5.05**	**2.14**	**3.25**	**0.00**	**15.06**	**0.00**	**2.54**	**3.91**	**1.73**	**20.12**
	LCI (95% CI)	*3.86*	*5.16*	*3.35*	*2.74*	*0.77*	*1.10*	*NA*	*3.32*	*NA*	*0.32*	*1.50*	*0.37*	*4.86*
	UCI (95% CI)	*7.99*	*10.93*	*6.65*	*8.04*	*4.13*	*6.57*	*NA*	*33.43*	*NA*	*7.01*	*7.56*	*4.08*	*43.00*
	**Ciprofloxacin**													
	**Prevalence**	**4.09**	**3.13**	**3.61**	**3.86**	**4.30**	**3.27**	**7.14**	**5.05**	**3.71**	**5.12**	**5.22**	**3.47**	**6.73**
	LCI (95% CI)	*2.51*	*1.50*	*2.31*	*1.86*	*2.28*	*1.17*	*0.94*	*0.16*	*0.12*	*1.45*	*2.32*	*1.28*	*0.23*
	UCI (95% CI)	*6.07*	*5.34*	*5.22*	*6.59*	*6.89*	*6.44*	*19.17*	*17.82*	*13.11*	*10.88*	*9.49*	*6.63*	*24.21*
	**Gentamicin**													
	Prevalence	**5.78**	**6.55**	**8.77**	**11.72**	**7.10**	**1.95**	**14.13**	**5.03**	**7.39**	**8.82**	**3.27**	**7.49**	**6.72**
	LCI (95% CI)	*3.85*	*4.18*	*6.69*	*8.08*	*4.47*	*0.40*	*4.17*	*0.11*	*0.93*	*3.58*	*1.14*	*4.05*	*0.23*
	UCI (95% CI)	*8.10*	*9.44*	*11.09*	*15.85*	*10.22*	*4.61*	*28.95*	*17.23*	*19.59*	*16.27*	*6.44*	*11.79*	*22.93*
	**Cefotaxime**													
	Prevalence	**8.90**	**5.93**	**14.60**	**12.52**	**9.58**	**5.87**	**10.82**	**19.94**	**11.17**	**7.60**	**10.45**	**7.98**	**13.31**
	LCI (95% CI)	*6.46*	*3.59*	*12.02*	*8.66*	*6.49*	*2.72*	*2.50*	*5.84*	*2.47*	*3.03*	*6.17*	*4.42*	*1.68*
	UCI (95% CI)	11.62	*8.72*	*17.46*	*16.92*	*13.33*	*9.97*	*24.24*	*38.95*	*24.58*	*14.53*	*15.66*	*12.33*	*34.91*
	**Cefepime**													
	Prevalence	**8.82**	**5.88**	**14.57**	**12.44**	**3.25**	**1.85**	**0.00**	**5.18**	**0.00**	**5.24**	**2.63**	**2.29**	**6.47**
	LCI (95% CI)	*6.42*	*3.66*	*11.92*	*8.77*	*1.54*	*0.37*	*NA*	*0.18*	*NA*	*1.54*	*0.74*	*0.69*	*0.17*
	UCI (95% CI)	*11.44*	*8.72*	*17.42*	*16.81*	*5.62*	*4.54*	*NA*	*17.85*	*NA*	*11.05*	*5.69*	*4.91*	*22.69*
	**Nalidixic A**													
	Prevalence	**11.97**	**12.13**	**10.66**	**10.12**	**11.72**	**12.86**	**7.19**	**15.29**	**3.72**	**12.67**	**14.27**	**10.95**	**20.07**
	LCI (95% CI)	*9.25*	*8.74*	*8.30*	*6.74*	*8.26*	*8.12*	*0.82*	*3.64*	*0.12*	*6.42*	*9.22*	*6.81*	*4.48*
	UCI (95% CI)	*14.99*	*15.80*	*13.15*	*14.09*	*15.60*	*18.42*	*19.01*	*33.92*	*12.69*	*20.60*	*20.54*	*16.01*	*43.16*
	**Streptomycin**													
	Prevalence	**73.58**	**82.84**	**76.45**	**75.38**	**75.88**	**74.16**	**46.58**	**75.03**	**51.79**	**63.23**	**75.95**	**79.25**	**80.37**
	LCI (95% CI)	*69.61*	*78.52*	*72.82*	*70.03*	*70.89*	*67.10*	*28.89*	*54.36*	*33.77*	*52.39*	*68.88*	*72.93*	*57.46*
	UCI (95% CI)	*77.45*	*86.63*	*79.73*	*80.45*	*80.74*	*80.67*	*65.24*	*90.65*	*69.71*	*73.22*	*82.42*	*84.88*	*95.46*
	**Sulphonamide**													
	Prevalence	**78.36**	**84.74**	**74.45**	**75.86**	**79.07**	**78.15**	**82.17**	**65.11**	**85.32**	**73.34**	**78.49**	**79.99**	**93.54**
	LCI (95% CI)	*74.61*	*80.89*	*71.07*	*70.57*	*74.38*	*71.45*	*66.22*	*44.45*	*69.85*	*63.31*	*72.02*	*73.77*	*77.13*
	UCI (95% CI)	*81.82*	*88.41*	*77.73*	*80.93*	*83.51*	*84.10*	*93.89*	*83.80*	*95.49*	*82.28*	*84.56*	*85.49*	*99.86*
	**Tetracycline**													
	Prevalence	**33.65**	**45.17**	**45.42**	**44.04**	**33.59**	**32.31**	**21.43**	**60.34**	**7.38**	**31.61**	**29.23**	**37.35**	**66.52**
	LCI (95% CI)	*29.53*	*39.92*	*41.48*	*38.08*	*28.28*	*25.31*	*8.74*	*37.90*	*1.01*	*21.97*	*22.24*	*30.37*	*42.15*
	UCI (95% CI)	*37.89*	*50.72*	*49.21*	*50.04*	*39.08*	*39.91*	*38.71*	*80.29*	*19.58*	*41.97*	*36.74*	*44.61*	*87.24*
	**Trimethoprim**													
	Prevalence	**34.87**	**55.80**	**42.43**	**42.57**	**36.19**	**31.54**	**31.94**	**45.33**	**18.72**	**34.24**	**29.85**	**39.51**	**53.24**
	LCI (95% CI)	*30.76*	*50.38*	*38.61*	*36.77*	*30.70*	*24.46*	*16.52*	*24.17*	*6.72*	*24.44*	*22.88*	*32.48*	*28.98*
	UCI (95% CI)	*39.25*	*61.38*	*46.34*	*48.81*	*41.88*	*39.18*	*49.53*	*67.26*	*35.31*	*45.04*	*37.46*	*46.74*	*76.57*

		Wildlife	Environmental	Human	Livestock
**Diversity Indices (Alpha)**	**PR (profile richness)**				
	Median	**71**	**93**	**68**	**79**
	LCI (95%)	*63*	*NA*	*62*	*70*
	UCI (95%)	*78*	*NA*	*73*	*88*
	**SE (Shannon entropy)**				
	Median	**31.18**	**43.14**	**33.87**	**36.02**
	LCI (95%)	*26.84*	*NA*	*30.7*	*30.36*
	UCI (95%)	*36.08*	*NA*	*37.06*	*42.5*
	**SD (Simpson diversity)**				
	Median	**16.01**	**20.67**	**22.01**	**18.74**
	LCI (95%)	*13.51*	*NA*	*17.99*	*15.89*
	UCI (95%)	*19.09*	*NA*	*22.31*	*22.55*
	**BP (Berger-Parker)**				
	Median	**8.54**	**11.5**	**11.97**	**11.35**
	LCI (95%)	*7.05*	*NA*	*10.56*	*9.61*
	UCI (95%)	*10.93*	*NA*	*13.62*	*13.29*

Table S5. Median values for each epidemiological compartment (wildlife, environmental, human, livestock) for four diversity indices (PR, SE, SD, BP) of phenotypic antimicrobial resistance. Environmental samples were resampled from, and as such 95% CIs were not calculated for this compartment. Wildlife had a statistically lower diversity across all levels of Alpha than all other compartments (P=0.00014).

A)

		Wildlife	Environmental	Human	Livestock
**Assyptotic Richness Estimator**	**Chao2**				
	Estimate	272.54	349.89*	184.73	416.06
	LCI (95%)	*244.63*	*304.99*	*204.86*	*378.1*
	UCI (95%)	*300.45*	*394.78*	*164.59*	*454.02*
	**ICE**				
	Estimate	163.67	240.57	116.38	236.91
	LCI (95%)	*NA*	*NA*	*NA*	*NA*
	UCI (95%)	*NA*	*NA*	*NA*	*NA*
	**Jacknife**				
	Estimate	170.85	160.7	127.85	240.84
	LCI (95%)	*NA*	*NA*	*NA*	*NA*
	UCI (95%)	*NA*	*NA*	*NA*	*NA*

B)

Compartment	t	T	S_{obs}	S_{est}	Q_1	Q_2	q_0	g=1	g=0.85	g=0.8
Wildlife	440	440	105	273	66	13	0.15	8848	1572	1251
Environment	236	236	93	350	68	9	0.29	7464*	1410*	1154*
Humans	310	310	80	185	48	11	0.15	5000	896	702
Livestock	585	585	145	416	96	17	0.16	13979	2421	1947

Table S6. A) Asymptotic estimates for the total number of antibiograms in each epidemiological compartment. Red, Chao 2 asymptotic estimate; *, estimator did not show asymptotic behaviour. B) Estimated sampling effort for each epidemiological compartment. Abbreviations are: t, number of samples collected; T, total number of incidences; S_{obs}, observed species richness; S_{est}, estimated asymptotic species richness, based on the Chao2 estimator; Q1, the number of species represented by exactly one sample (‘‘uniques’’); Q2, the number of species represented by exactly two samples (‘‘duplicates’’); q0, the probability that the next observed sample contains a species new to the survey (i.e., the proportion of species in the next sample that are new to the survey); g, target fraction of S_{est} that is to be reached. The entries in each ‘‘g’’ column represent the number of additional samples required to detect that proportion of S_{est}. The estimators for environment did not show asymptotic behaviour, and as such values marked with * are interpreted with caution.

Model: MDR carriage in wildlife	Estimate	Std. Error	z value	P-value
Intercept	-1.6578	0.7756	-2.137	0.033
Avian_VertFishScav	2.3775	0.9961	2.387	0.017
Avian_Invertebrate	1.3414	0.8217	1.632	0.1
Avian_Omnivore	2.0119	0.8494	2.369	0.018
Avian_Seed	1.842	0.7926	2.324	0.02
Bat_Fruit	2.3151	0.976	2.372	0.018
Bat_Invertebrate	0.978	0.9477	1.032	0.3
Rodent_Omnivore	1.524	0.7997	1.906	0.057
MEM1	-0.2877	0.1212	-2.373	0.018

Model: AMR-profile length in wildlife	Estimate	Std. Error	z value	P-value
Intercept	1.075	0.0429	25.046	<0.0001
Bat	-0.0971	0.1294	-0.75	0.45
Rodent	-0.1613	0.0669	-2.412	0.016
MEM1	-0.1192	0.0357	-3.34	0.00084
MEM8	0.1034	0.0333	3.101	0.0019
MEM10	0.0728	0.035	2.078	0.038
MEM19	-0.0792	0.0325	-2.438	0.015
MEM25	-0.064	0.0304	-2.104	0.035
MEM27	-0.0827	0.03	-2.761	0.0058
Laboratory (UoN)	0.2253	0.0724	3.112	0.0019

Table S7. Estimated regression parameters, standard errors, z-values and P-values for the Binomial and Poisson generalised linear mixed effects models, which compare the likelihood of different wildlife functional groups carrying multi-drug resistant (MDR)-*E. coli*, and the length of *E. coli* antibiotic profiles across wildlife functional groups. MEMs indicate the spatial scales across which variation in MDR carriage or profile length occurs. Increasing MEM numbering indicates increasingly finer spatial scales.

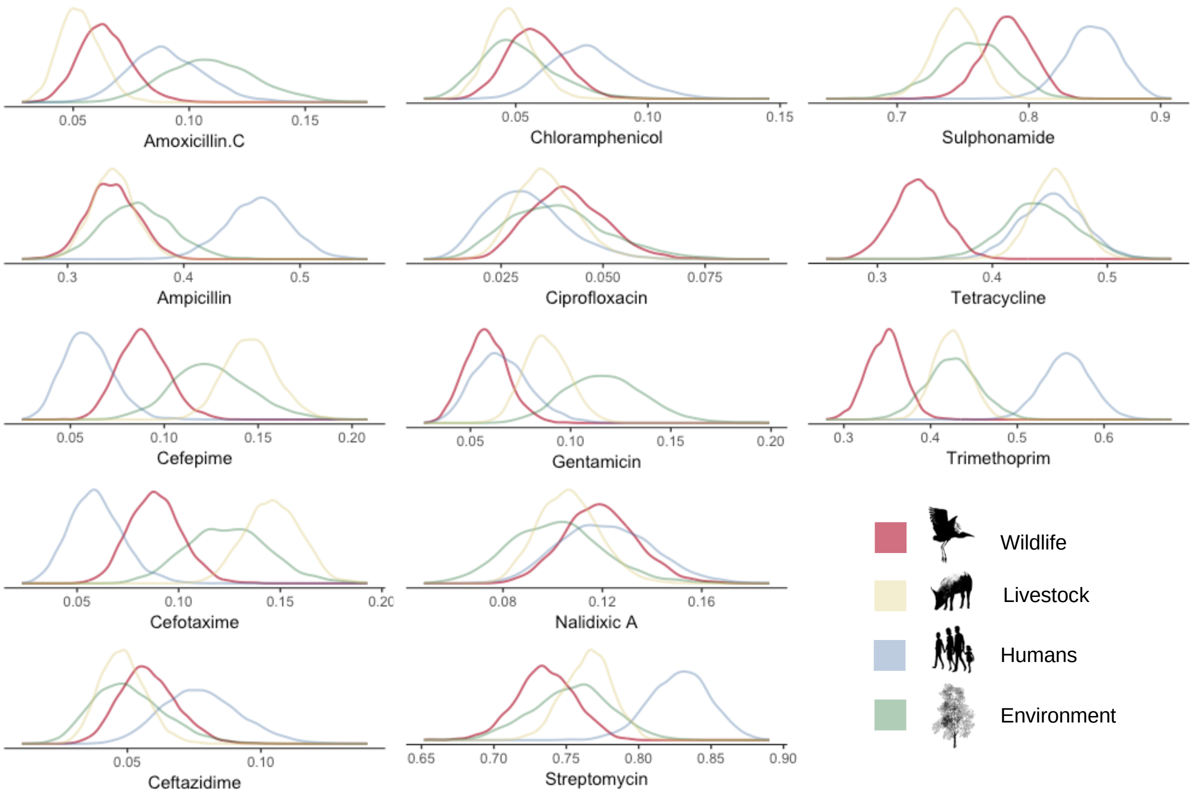

Figure S1. Bayesian posterior distributions for the prevalence (x-axis) of 13 antibiotics in wildlife (red), livestock (green), human (blue) and environmental (yellow) samples.

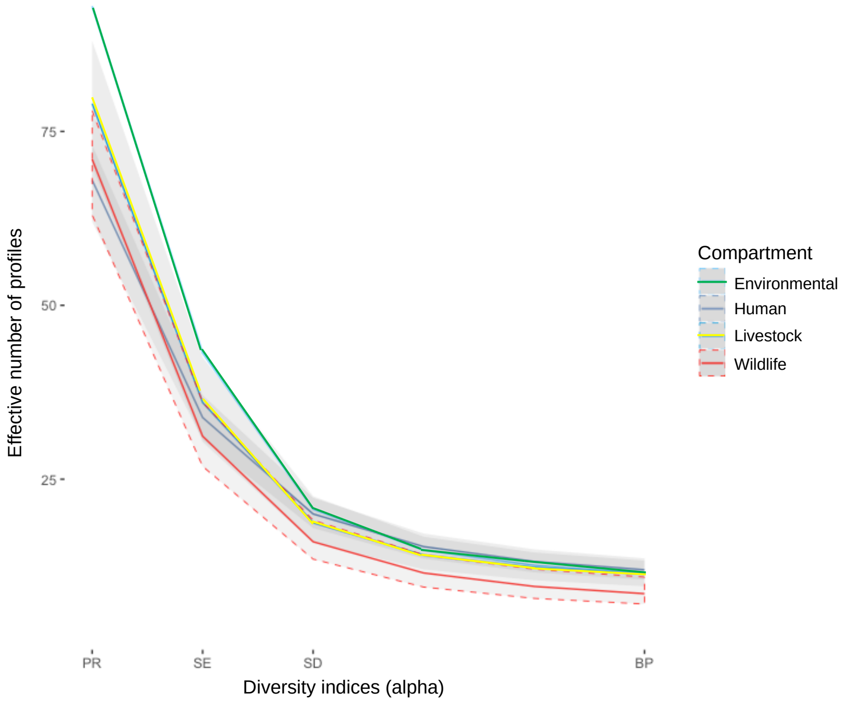

Figure S2. Diversity of AMR profiles (measured as the effective number of AMR profiles) across four diversity indices, for environmental, human, livestock and wildlife compartments. Shaded areas (grey) represent 95% CI intervals around the median for each line. Dashed line (red) highlights 95% CI for wildlife. PR, profile richness; SE, Shannon entropy; SD, Simpson diversity; BP, Berger-Parker. Ranges for Alpha were significantly lower in wildlife than for all other compartments.

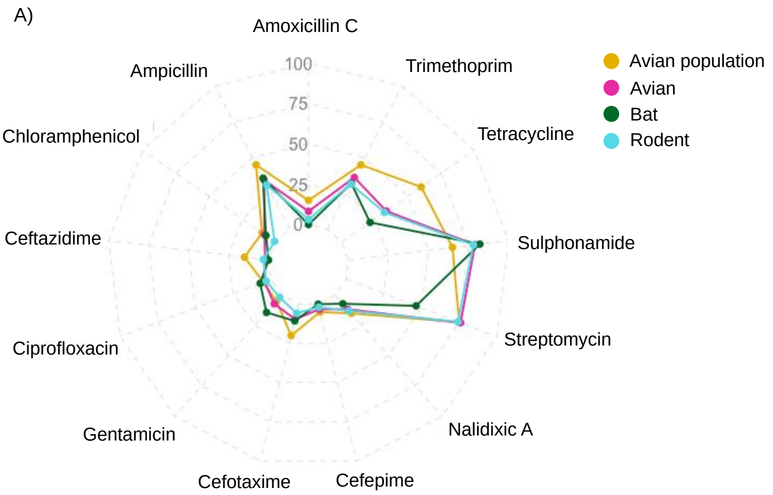

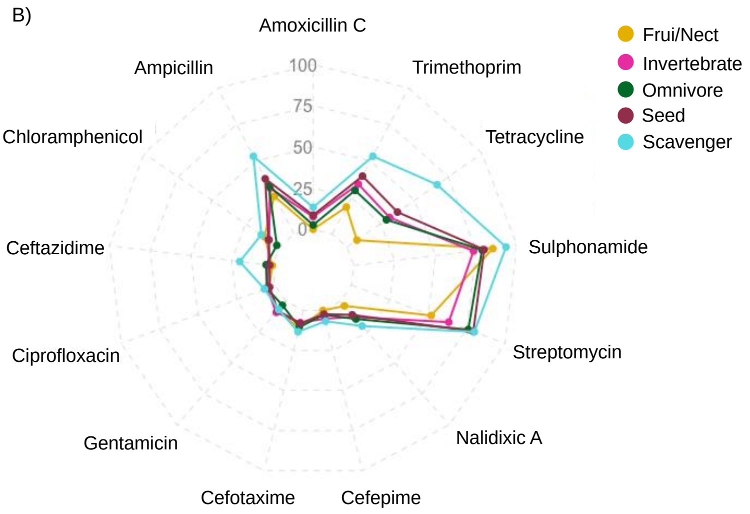

Figure S3. Radar plots showing differences in prevalence for resistance of *E. coli* to the 13 antibiotics tested for between A) wildlife taxonomic groups (avian populations, birds, bats and rodents), and B) wildlife function groups stratified by feeding ecology.

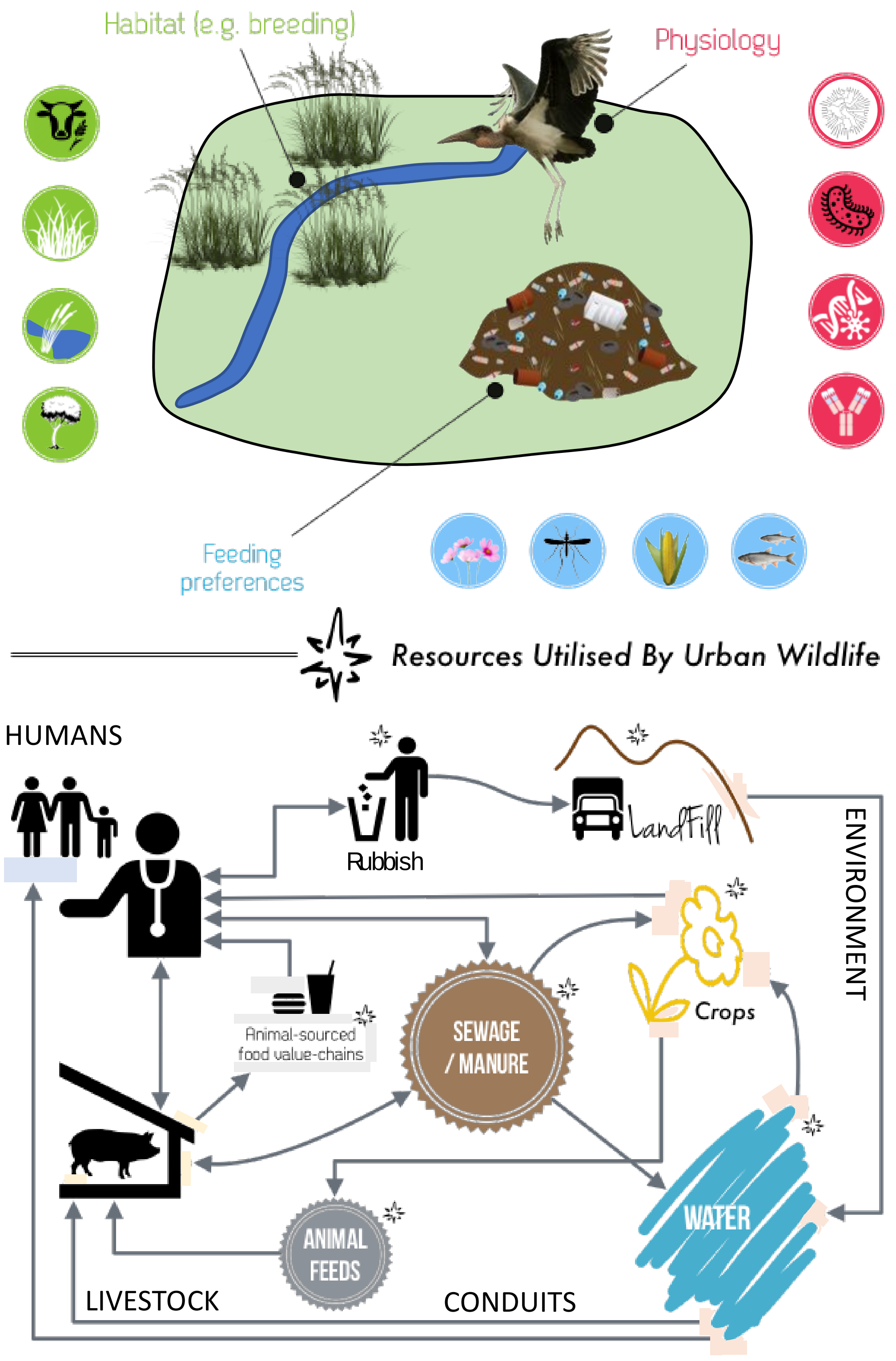

Figure S4. Graphic exploring the role played by urban wildlife in exposure to and dissemination of antimicrobial resistance (AMR) determinants. The top panel describes three main groups of determinants that govern the carriage, exposure and dissemination of AMR in urban wildlife. The bottom panel depicts the movement of AMR determinants in urban ecosystems. Stars denote compartments that urban wildlife are likely to utilise as resources, and thus potential routes of wildlife exposure to AMR.

Supplementary Methodological Appendix

Study Design

The UrbanZoo project, based in Nairobi, Kenya from 2012-2017, aimed to utilise a landscape genetics approach to understanding the movement and sharing of pathogens in a major developing city. A key component of this project, within which this study was nested, was the '99 household project', which focused on informal livestock keeping practices in urban households as a route of zoonotic disease emergence in humans. As such, households were selected with the aim of maximising the spatial distribution and diversity of livestock keeping practices across Nairobi, and were chosen to capture three main criteria: socio-economic diversity, population distribution and livestock keeping practices. Geospatial mapping data, generated as part of a technical report produced by Institut Français de Recherche en Afrique (IFRA), was used to identify 17 classes of residential neighbourhood in Nairobi based on physical landscape attributes, which were subsequently verified by 817 household questionnaires[1]. Each of the 17 classes of neighbourhood were then ranked by average income and reduced into seven wealth groups. Administrative sublocations were mapped onto each wealth group, identifying a total of 70 possible sublocations, for which dominant wealth groups were calculated by extracting the proportion of population belonging to each neighbourhood class within the sub-location boundaries (Table SM1). A total of 33 sublocations were selected to be included in the study, with the number of sublocations belonging to each wealth group chosen proportionately to the population density and the variety of neighbourhood classes in each of the seven wealth groups. Final selection of individual sublocations was aimed at maximising areas with high livestock densities, whilst ensuring coverage of other neighbourhood classes and geographical spread.

Field sampling was conducted between September 2015 and September 2016. For each sublocation, three geographical points were selected at random within the dominant housing type. The order in which sublocations were visited was randomised. Local officials assisted in the recruitment of a household closest to each geographical point, to obtain two livestock keeping and one non-livestock keeping household per sublocation (a total of 99 households, 66 of which kept livestock). Households had to meet strict inclusion criteria of keeping either large ruminants (cattle), large monogastrics (pigs), small ruminants (goats/sheep), small monogastrics (poultry/rabbits), or no livestock species. To ensure an equal sample of both cattle and pig-keeping households, the combination of livestock keeping households represented in each sublocation was randomised, and had to consist of either large ruminant and small monogastric, or large monogastric and small ruminant species. For sublocations in which households keeping large ruminant or large monogastric species were absent, a replacement household keeping either small monogastic or small ruminant species was recruited. Rodents and scavenging birds were sampled from abattoirs in Nairobi, which were selected as part of a separate value chain study conducted as part of the wider UrbanZoo project[2].

Data Collection

Sampling. Two dedicated field teams were responsible for collecting data on humans, livestock and wildlife in each household, consisting of veterinarians, animal health technicians and clinicians. Informed consent was obtained from human participants, who were invited to submit a stool sample. Up to 20 rectal swabs were obtained from livestock species present in the household (ensuring that all species were represented in the sample). Rodents, bats, birds, small carnivores and non-human primates (NHPs) were all targeted for wildlife sampling. Rodents were trapped using medium-sized (23 cm x 7.5 cm x 9 cm) Sherman live traps (H. B. Sherman Traps Inc., Tallahassee, FL) or Victor lethal traps (Woodstream Corp., Lititz, PA) that were baited with dried fish, placed against walls throughout the household and livestock keeping facilities, and left in place for three nights. Where possible, traps were set in each household for all trapping nights and checked daily. Mist nets were set at dawn to trap birds, with nets being positioned outside the house and around livestock keeping facilities. For household compounds in which bat activity was deemed likely (as judged based on the presence of fruiting trees and/or 'flyways'), mist nets were set at dusk and monitored for two hours. Where security conditions permitted, a remote bat detector (Song Meter ZC, Wildlife Acoustics, Inc.) was placed in each household compound for a single night, to monitor ultrasonic bat activity. If members of the household reported seeing small carnivores (such as mongoose) then Tomahawk cage traps (Tomahawk Live Trap Company, Tomahawk, Wis.) were set, baited with chicken and monitored regularly for a maximum of three days. Where NHP activity was reported at a household, wire-mesh live-capture traps were pre-baited with bananas for a minimum of three days. Traps were then set, and monitored regularly for a maximum of three days. Due to large variation in the size of household compounds, trapping effort (i.e. number of traps/mist nets placed per trapping session) was maintained such that it was proportional to the size of the household compound. The number of wildlife and livestock sampled are presented in Table SM2.

Once caught, all birds, and all but two bats caught per trapping session, were live-sampled in the field under manual restraint, before being released unharmed. Morphometric data was collected for identification purposes, and a suite of biological samples (including faeces if available, or a rectal/cloacal swab) were collected from each animal. All live rodents (except for individuals belonging to the genus *Cricetomys*, which were live-sampled under anaesthesia) and up to two bats caught per trapping session were transferred back to a biosafety level three (BSL3) laboratory, and humanely euthanised by cardiac puncture under isoflurane anaesthesia. Species identification was based on morphometric data. A full post-mortem examination was then performed, with fresh faeces being collected from the rectum. Rodents caught in lethal traps were also necropsied in the laboratory following the same protocols. Faecal samples were collected non-invasively from small carnivores, by keeping them in the trap for a maximum period of twelve hours. NHPs were anaesthetised where trapped, using a combination of Medetomidine and Ketamine (under the supervision of a Kenya Wildlife Service veterinary officer), and morphometric data and a suite of biological samples (including faeces if available, or a rectal swab) were collected from each animal. The primate was carefully monitored throughout, and anaesthesia reversed using Atipamezol. Carnivores and NHPs were released unharmed at an appropriate time of day, from the same location at which they were trapped.

Pooled samples were collected from scavenging and water birds, by positioning tarpaulins under roosting sites located in tress in proximity to household compounds. Clean tarpaulins were either left out overnight (or monitored over the space of ~30 minutes during the day), and any fresh faeces that were present at the time of collection were pooled into a single sample. By restricting pooled sample collection to different sections of tarpaulin, we ensured that individual birds were not represented across multiple samples.

Outdoor environmental samples were collected from livestock pens and around the household compound using boot socks[3]. Water samples were collected from puddles and flowing water (i.e. rivers), using falcon tubes and modified Moore swabs respectively[4]. Moore swabs were formed from a tightly rolled piece of dishcloth folded horizontally in half several times and tightly rolled into a cylindrical shape. These were suspended in running water using fishing line for roughly 24 hours. Questionnaire and sample data was recorded using Open Data Kit (ODK) Collect software[5], on electronic tablets, and uploaded to databases held on servers at the International Livestock Research Institute (ILRI).

Household questionnaires. A nominated member of each household completed a questionnaire, detailing *i)* livestock ownership, management, sourcing, sales and antimicrobial use, and *ii)* household composition and socio-economic data. Abundance (counts) of livestock species and humans were derived from this data for each household. Household composition and socio-economic data were used to generate 'wealth' and 'ruralness' indices for each household sampled[6]. These indices were calculated based on methods used to create the Demographic and Health Surveys (DHS) wealth index, which is derived from a Principal Component Analysis (PCA) of easily measurable households assets (such as access to water, construction materials and ownership of livestock)[7]. A modification was made to the original set of household assets included in the DHS index to better capture household variation in Nairobi. All field data was recorded using Open Data Kit (ODK) Collect software [5], on electronic tablets, and uploaded to databases held on servers at the International Livestock Research Institute (ILRI).

Land-use classification. Nairobi is characterised by a large variety of land use. Land use comprises the biotic and abiotic niches within which hosts exist, and was classified for each household. The boundary of each household compound was drawn in ArcMap, and a 30m buffer created around the perimeter of each compound to represent the landscape surrounding it. A buffer of 30m was chosen to reflect home range of common urban rodent species (*Mus* and *rattus* spp., estimates of which vary from 1m to 30m)[8,9]. Visual classification of land-use types within the compound and buffer area were conducted at 1:500 scale on a 1m resolution ESRI World Imagery satellite-image available in ArcGIS 10.5 (ESRI). Characterisation of ecological characteristics along a perimeter around the household compound was considered as important, because the ecological setting within which the household exists extends beyond the boundaries of the compound. The extent to which this influential area of habitat outside the compound extends is unknown, and as such it was standardised across study sites. Within the boundary, the areas of nine different land-use types were visually identified and sketched as polygons; water-body, wetland, crops, mature trees, shrubs, grassland, bare ground, artificial ground and rubbish (descriptions for each of these are summarised in Table SM3). The total area of classified land-use types at each site were calculated and expressed as proportions. All classification was undertaken by J.M.H. who was familiar with the landscape at each site, and subsequently ground-truthed by revisiting sites.

Supplementary Statistical Appendix S2

Analytical approach

Comparison of prevalence, multidrug resistance and antibiogram diversity between wildlife, livestock, human and environmental compartments. A Bayesian analysis framework with MCMC was used to compare prevalence of resistance to 13 antibiotics between epidemiological compartments. This was applied using JAGS via the R package 'R2jags' [10,11]. A burn-in of 4000 iterations, three chains, and a thinning rate of 10 and 15,000 iterations for each posterior distribution was used. Diffuse normal priors $N(0,100^2)$ were used for the Bernoulli distribution, and the prior densities were plotted. Variation in prevalence between compartments was assessed by comparing posterior densities. Generalised Linear Mixed Effects Models (GLMMs) were developed to test whether MDR-*E. coli* carriage and AMR profile length differed between host compartments. The response variable for MDR-*E. coli* carriage was coded from 0 to 1, and antibiogram length was measured from 1 to 13; as such, a binomial distribution with logistic link function and Poisson distribution were used, respectively.

To assess how wildlife AMR profile diversity was distributed across all four epidemiological compartments (wildlife, livestock, human, environment), diversity was compared between compartments using four ecological measures of diversity related to Rényi's measures of generalized entropy. This approach is described by Mather et al. [12], where the exponential of Rényi's entropy (Dα) estimates the effective number of species (AMR profiles in this case), and α represents a scale parameter, along which profile richness and relative abundance of profiles are weighted differently. The four diversity indices that were compared across compartments sit at different levels of α: at D0, profile richness (PR) is a count of AMR profiles (which ignores relative abundance, thus considering rare and common profiles equally); at log(D1), Shannon entropy (SEn) is the probability of any two isolates drawn at random having the same profile; at 1/D2 Simpson diversity (SD) is the relative abundance of each profile; and at 1/D∞ the Berger-Parker diversity (BP) is the proportion of the most common profile in the sample. Adjustments were made to each diversity measure to account for variation in sample size between compartments by resampling all compartments to the sample size of the smallest compartment (environment), with replacement. Bootstrapping 1000 times provided a median value with confidence intervals (CIs) for each of the diversity measures. For each index, the diversity index differences were compared between compartments using a Kruskal-Wallis test with Holm adjustment for multiple comparisons to compare ranges of α.

Methods adapted from the community ecology literature were used to extend the comparison of phenotypic diversity between epidemiological compartments by estimating the number of undetected AMR profiles in each epidemiological compartment. This approach is similar to that of Anthony et al [13], who used comparable approaches to estimate mammalian viral diversity. Rarefaction and species accumulation curves were generated from the incidence data for all 13 antibiotics. The 'exact' accumulation method and non-linear regression models were used to estimate profile-area relationships, and thus the shape of the species accumulation curves. An appropriate non-linear model for each compartment was selected by subsampling half of the data, and extrapolating to the total number of samples in each compartment. Models with the closest predictions to the correct number of species were used. Sampling to the asymptote of these curves would reveal the total number of profiles in each compartment, but at prohibitive effort and costs. As such, Chao2, ICE and Jackknife incidence-based statistical methods were used to estimate the minimum total profile richness in each compartment from the data, by looking at frequencies of phenotype occurrence in collections of individuals. These nonparametric estimators are considered more robust than model-based estimates, and particularly suited to the purposes of comparison between compartments, because they make no assumptions of distributions underlying species detection rates, which may differ between assemblages [14]. Rarefaction and species accumulation were performed in the R package 'vegan' [15], and richness estimates were computed in the R package 'fossil' [16]. To consider the implications for surveillance, methods from Chao et al [17] were followed to estimate the sampling effort required to detect a given proportion of the total AMR profiles estimated for each compartment.

Role of wildlife functional ecology in AMR-*E.coli* carriage. A Bayesian analysis framework, as described above, was used to estimate prevalence of resistance to 13 antibiotics between wildlife taxa. Ecological traits considered potentially important factors for exposure of wildlife to AMR were sourced from metadata and published sources (see Table S3). These included taxonomic group (avian, bat, rodent), feeding ecology (food source and canopy strata) [18], home range, and association with water [19]. Home range estimates for all species except bats were calculated by allometric scaling of body weight [20]. Scaling factors published for functionally different mammals and birds by Ottoviani et al [21] were used, and species mean body weights were either collected during sampling, or sourced from published datasets when unavailable [8,22]. Two GLMMs were developed to investigate variation in the likelihood of multidrug resistant (MDR)-*E. coli* carriage and length of antibiograms in wildlife, as determined by these functional traits. The response variable for MDR-*E. coli* carriage was coded from 0 to 1, and antibiogram length was measured from 1 to 13; as such, a binomial distribution with logistic link function and Poisson distribution were used, respectively. For each set of response and explanatory variables, data

exploration was carried out following the protocol described in Zuur et al [23]. Data exploration revealed marked correlation between several of the explanatory variables, and as such, taxonomic groups and feeding niches were either combined or considered separately, and association with water was not considered as a covariate. Spatial structure was captured using Distance-based Moran's eigenvector maps (dbMEM), and the dependency structure of each model was assessed by fitting random-intercept models to the response data. The full models including all covariates are depicted in Figure SM1.

AMR exchange between wildlife, livestock and humans within households. A separate set of GLMMs were developed to investigate household-level risk factors for the likelihood of MDR-*E. coli* carriage in select urban wildlife with synanthropic traits (rodents and seed-eating birds). A set of anthropogenic and ecological covariates capturing AMR-*E. coli* carriage in livestock and humans, livestock keeping practices, land use within households and ranging behaviour of wildlife were derived from metadata and published sources (Table S3). The proportion of cropland within each household was mapped by visual classification using ArcGIS, and subsequently ground truthed within each household (for further details see Chapter 2.5). Separate GLMMs with binomial distribution and logistic link function were fitted for rodents and seed-eating birds, using the same set of covariates. The response variable, MDR-*E. coli* carriage, was coded from 0 to 1. For each set of response and explanatory variables, data exploration was carried out following the protocol described in Zuur et al [23]. Spatial structure was captured using db-MEMs and the dependency structure of each model was assessed by fitting random-intercept models to the response data. The full models including all covariates are depicted in Figure SM1.

Missing Data. Missing data consisted of *i)* a single household that declined to take part in the study, but for which wildlife samples were collected, and *ii)* unidentified birds and rodents, which could not be accurately assigned to a functional group. This data was treated as Missing Completely at Random (MCAR), and isolates were subsequently removed from the respective analyses. For household-level comparative analyses between wildlife, livestock and humans, wildlife isolates collected from the household that declined to participate were removed from the analysis (n=7). For comparative analyses between wildlife functional groups, isolates from animals that could not be assigned to a functional group were excluded (n=12).

Generalised linear mixed models (further details). Data exploration was used to test for and address potential statistical problems in the dataset that could inflate type I and type II error. Following protocols described by Zuur *et al.* [23], the main sources of error tested for in the development of each model were the influence of outliers in the response or explanatory variables, collinearity between explanatory variables, and zero-inflation (the number of zeros in the data). For each model, relationships between all sets of explanatory covariates were assessed using multi-panel pairwise scatterplots, Pearson correlation coefficients, and variance inflation factors (VIF). To reduce the influence of collinearity on model parameter estimates, variables with high VIFs were sequentially dropped, until all VIFs were smaller than a preselected threshold of 3 [23]. Only these variables were carried forward to the full model, and subsequent model selection procedures. Cleveland dotplots were used to identify outliers in response and explanatory variables. Outlying data points in response variables were addressed by selecting an appropriate probability distribution for use in the model (e.g. using a Poisson distribution for count data). Where outlying data points were present in explanatory variables (and were not believed to be true measurement errors), log base 10 or square-root transformations were applied to the variable in question.

GLMMs were constructed based on hypothesis-driven ecological reasoning, in which biological understanding of the underlying system was used to build a global model by selecting variables *a-priori*. This dataset consists of multiple observations within each household, which are nested within sublocations. As such, where generalised mixed effects models were used, random effects household, sublocation, or both were included (the dependency structure of each model was assessed separately by fitting random-intercept models to the response data, and comparing the intra-class correlation coefficient [ICC, latent variable method [24]]). To account for the fact that antimicrobial sensitivity testing of wildlife samples was split between two laboratories, the laboratory at which each sample was tested was included as a covariate in each model. Sampling was carried out over the course of a year, and as such, temporal trends in the dataset were assessed by plotting mean MDR-*E. coli* carriage against the week in which samples were collected (Figure SM2).

Optimal models were constructed using stepwise, backwards elimination from the full model based upon Akaike information criteria (AIC). Significance of model terms were tested by the maximum likelihood test, and the fit of each model was reported as marginal regression coefficients of multiple determination (marginal R^2) where possible. Model assumptions were verified by plotting residuals versus fitted values, and by assessing models for overdispersion. Non-linear relationships were checked by fitting a generalized additive model (GAM) between the response and explanatory variables, featuring a nonlinear smoother, in R package 'mgcv'[25]. The residuals were

also assessed for spatial dependency by plotting them against geographic coordinates, and examining the results of a semivariogram in R package 'gstat' [26].

Modelling spatial structure with dbMEMs (further details). Urban environments and their host communities are spatially heterogeneous. Understanding spatial structures present across the urban landscape and in these communities, could indicate the underlying processes that have created them, and as such, it is important to capture this spatial structure in statistical models. In complex ecological and epidemiological systems, spatial structure operates across multiple scales, and a single response variable can display structure at more than one spatial scale [27]. To address this, a statistical method called Distance-based Moran's eigenvector maps (dbMEM) was used to represent spatial structure across all scales in the models in this study [28]. This approach begins with identifying the scales at which spatial structure (autocorrelation) is present in the response variable. The dbMEM base functions (eigenvectors) which represent structure in the response variable, are generated through a PCoA performed on a matrix of geographic distances between sites (households). Linear trends are tested for, and, if identified in the data, spatial detrending is applied before conducting dbMEM analysis (as recommended by Boccard et al [28]). Eigenvectors modelling positive spatial correlation are extracted, and regressed against the response variable to determine a set of significant dbMEM base functions modelling spatial structure in the response variable. These base functions can then be included as explanatory variables in a global model, or their variance can be removed from the model if spatial structure is deemed a 'nuisance variable'. To make them ecologically meaningful, dbMEM base functions can be split into arbitrary groups representing different spatial scales. Generally, base functions can be ordered from broad- to fine-scale as their number increases (e.g. Figure SM3) [27].

**Characteristics of physical neighbourhood classes identified by IFRA study (adapted from Ledant *et al.* [1])**				**Urban Zoo Project re-classification**		
Tree cover	Defining characteristics	Neighbourhood description (housing type)	Average income	Wealth group	Possible sub-locations	Targeted sub-locations
> 13.5%	Detached housing with intense tree cover	Detached housing on very large plots *(>3000 m^2)*	39,890	1	8	3
		Detached housing on large plots *(400 - 3000 m^2)*	22,462	2	8	4
	Attached and semi-detached housing	Attached housing on medium plots *(<400 m^2)* with important tree cover	22,084	2		
3% < 13.5%	Apartment building	Apartment buildings *with gated space*	22,084	2		
	Attached and semi-detached housing	Higher standing row houses *(plot size > 190 m^2)*	13,352	3	5	3
		Lower standing row houses *(plot size < 190 m^2)*	6,153	4	3	3
<3%	Roof cover >50% tiles	Lower standing *apartment buildings*	6,153	4		
		New areas of dense *single housing* development	3,855	5	9	5
	Roof cover > 40% concrete	High density multi-storey buildings	3,855	5		
3% < 13.5%	Apartment building	Apartment buildings *with open access*	3,855	5		
	Peripheral areas	Peripheral areas with residential component *(mainly residential)*	3,855	5		
		Peripheral areas with rural component *(presence of agriculture)*	2,165	6	24	11
	Collective housing	Community housing *with gated space*	2,165	6		
		Community housing *with open access*	2,165	6		
<3%	Roof cover	New areas of low quality housing *(built-up area <37%)*	2,165	6		

Table SM1. The seven wealth groups used by the Urban Zoo Project, and the number of sub-locations with a dominant wealth group identified and selected in the Nairobi municipality. Reprinted with permission from Bettridge *et al.* [5].

Taxonomic group	No. of individuals sampled	No. of species sampled (wildlife)
avian	547	57
avian populations	26 samples (4 pools)	4
bats	44	6
bat populations	20 samples (4 pools)	2
cattle	68	-
carnivores	5	2
chickens	271	-
ducks	27	-
geese	21	-
goats	112	-
g-pigs	4	-
g-fowl	21	-
pigs	53	-
pigeons	14	-
primates	4	2
rabbits	50	-
rodents	148	6
sheep	26	-
turkeys	10	-

Table SM2: Number of individual wildlife and livestock sampled as part of the 99 households project

Land Use Class	Explanation
Water-body (environmental, biotic)	Natural water body (flowing or non-flowing)
Wetland (environmental, biotic)	Open wetland – reeds/rushes
Cropland (anthropogenic, biotic)	Row cops or other herbaceous crops (e.g. maize, coffee etc.)
Trees (environmental, biotic)	Trees (single or multiple), as determined by presence of a clear crown and evidence of shadow cast on ground (includes plantations of trees for commercial purposes [e.g. fruit])
Shrubs (environmental, biotic)	Shrubs, where able to distinguish from grassland and trees
Grassland (environmental, biotic)	Grass, pasture, herbaceous rangeland or bare-ground not serving a human purpose.
Bare ground (anthropogenic, abiotic)	Heavily compacted soil, serving human purpose (e.g. dirt road, playground)
Artificial (anthropogenic, abiotic)	Synthetic, man-made surface or object (including water-bodies) (e.g. tarmac road, cement, roof, swimming pool, water tank)
Rubbish (anthropogenic, abiotic)	Accumulation of human-derived waste

Table SM3: Land use classifications

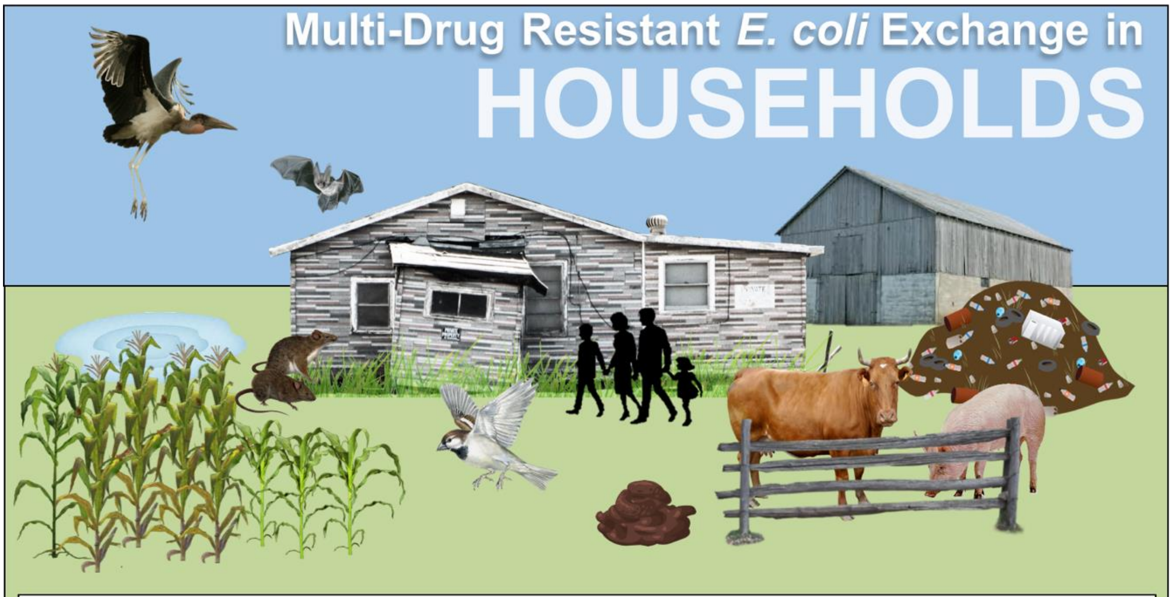

$$\mathrm{MDR}_j \sim \mathrm{Binomial}(\mu_j)$$
$$E(\mathrm{MDR}_j) = \mu_j$$
$$\frac{\log(\mu_j)}{1+\log(\mu_j)} = \text{Home Range}_i + \text{\%Cropland}_i + \text{Wealth Index}_i + \text{Total Cattle}_i + \text{Total Sheep}_i + \text{Total Goat}_i + \text{Total Pig}_j + \text{Total Indigenous Poultry}_j + \text{Total Lagomorph}_j + \text{Total Other Poultry}_j + \text{Total Humans}_j + \text{Garbage}_j + \text{Manure}_j + \text{Human ABG}_i + \text{Livestock ABG}_j + \text{Garbage}_j \times \text{Manure}_j + \text{Total Cattle}_j \times \text{Manure}_j + \text{Human ABG}_j \times \text{Garbage}_j + \text{Livestock ABG}_j \times \text{Garbage}_j + \text{Human ABG}_j \times \text{Manure}_j + \text{Livestock ABG}_i \times \text{Manure}_i + \text{Laboratory}_j$$

Figure SM1. Diagrammatic and mathematical representation of the full Binomial generalised linear mixed effects model (GLMM, before covariate selection) exploring variation in seed-eating bird and a rodent MDR-*E. coli* carriage, as a function of household-level ecological and anthropogenic covariates. ABG denotes antibiogram length.

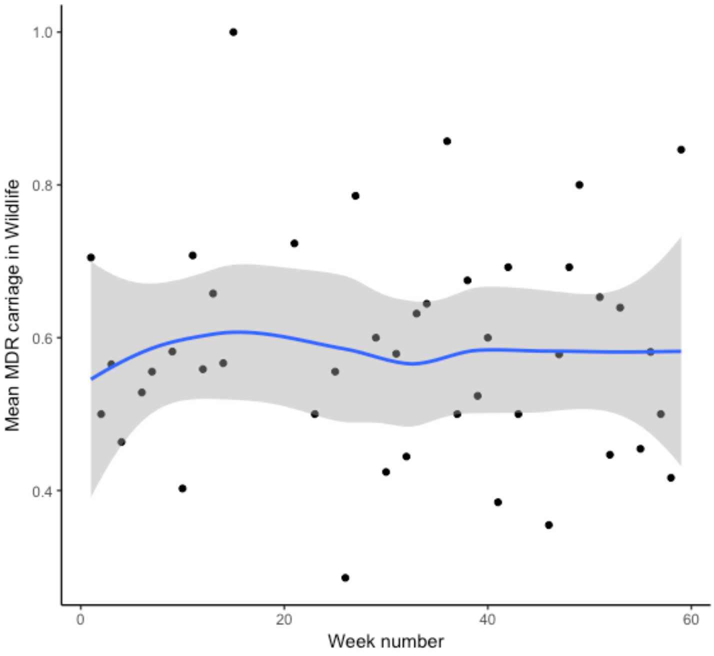

Figure SM2. Plot showing variation in mean multidrug resistant (MDR)-*E. coli* carriage in wildlife sampled per week, according to the week of the study in which sampling was conducted (blue line: mean, grey shaded area: 95% CI's around the mean).

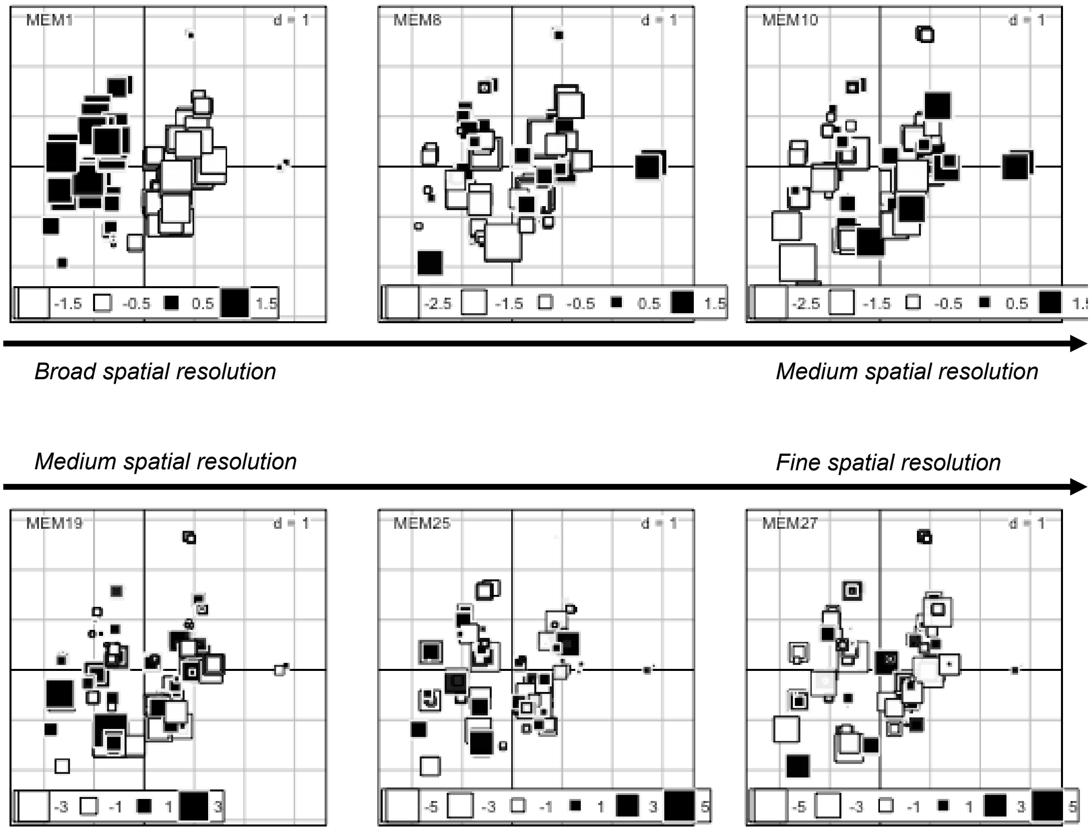

Figure SM3. Plot showing dbMEM eigenvectors modelling spatial variation in households at different scales across Nairobi. Increasing numeric magnitude of dbMEMs represents a gradient of broad to fine-scale spatial structure. As such, in this example MEM1, MEM8 and MEM10 model broad through to medium scale spatial resolution, and MEM19, MEM25 and MEM27 model medium through to fine-scale spatial resolution across Nairobi. Black and white blocks represent the GPS location of households.

References

1 Ledant M. Socio-Economical and Infrastrucural Mapping and Analysis of Nairobi: Technical Report. 2011.

2 Alarcon P, Fèvre EM, Muinde P, *et al.* Urban Livestock Keeping in the City of Nairobi: Diversity of Production Systems, Supply Chains, and Their Disease Management and Risks. *Front Vet Sci* 2017; **4**: 171.

3 Berghaus RDR, Thayer SGS, Law BF, Mild RM, Hofacre CL, Singer RS. Enumeration of Salmonella and Campylobacter spp. in environmental farm samples and processing plant carcass rinses from commercial broiler chicken flocks. *Appl …* 2013; **79**: 4106–14.

4 Sbodio A, Maeda S, Lopez-Velasco G, Suslow T V. Modified Moore swab optimization and validation in capturing E. coli O157:H7 and Salmonella enterica in large volume field samples of irrigation water. *Food Res Int* 2013; **51**: 654–62.

5 Hartung C, Anokwa Y, Brunette W, Lerer A, Tseng C, Borriello G. Open Data Kit: Tools to Build Information Services for Developing Regions. *Proc Int Conf Inf Commun Technol Dev* 2010; : 1–11.

6 Bettridge JM, Robinson TR, Hassell JM, *et al.* Soup-E.coli-field logistics-epi-and-genomics: A sampling strategy to capture bacterial diversity in a changing urban environment. In: Society for Veterinary Epidemiology and Preventative Medicine, Inverness, March 29-31. 2017.

7 Johnson K, Oscar Rutstein S. The DHS Wealth Index. In: DHS Comparative Reports No. 6. Bethesda, Md: ORC Macro, 2004.

8 Jones KE, Bielby J, Cardillo M, *et al.* PanTHERIA: a species-level database of life history, ecology, and geography of extant and recently extinct mammals. *Ecology* 2009; **90**: 2648–2648.

9 Lambert MS, Quy RJ, Smith RH, Cowan DP. The effect of habitat management on home-range size and survival of rural Norway rat populations. *J Appl Ecol* 2008; **45**: 1753–61.

10 Plummer M. JAGS: A program for analysis of Bayesian graphical models using Gibbs sampling. 2003.

11 Su YS, Yajima M. R2jags: A Package for Running Jags from R. 2012.

12 Mather AEAE, Matthews L, Mellor DJDJ, *et al.* An ecological approach to assessing the epidemiology of antimicrobial resistance in animal and human populations. *Proc Biol Sci* 2012; **279**: 1630–9.

13 Anthony S, Epstein J, Murray K. A Strategy To Estimate Unknown Viral Diversity in Mammals. *MBio* 2013. DOI:10.1128/mBio.00598-13.Editor.

14 Chao A, Chiu C. Species Richness: Estimation and Comparison. In: Chichester, ed. eLS. John Wiley & Sons, 2016: 1–11.

15 Okansen J, Blanchet GF, Friendly M, *et al.* Community Ecology Package. 2017.

16 Vavrek MJ. Palaeoecological and Palaeogeographical Analysis Tools. .

17 Chao A, Colwell RK, Lin CW, Gotelli NJ. Sufficient sampling for asymptotic minimum species richness estimators. *Ecology* 2009; **90**: 1125–33.

18 Wilman H, Belmaker J, Simpson J, de la Rosa C, Rivadeneira MM, Jetz W. EltonTraits 1.0: Species-level foraging attributes of the world's birds and mammals. *Ecology* 2014; **95**: 2027.

19 IUCN. The IUCN Red List of Threatened Species. 2017.

20 Jetz W, Carbone C, Fulford J, Brown JH. The scaling of animal space use. *Science* 2004; **306**: 266–8.

21 Ottaviani D, Cairns SC, Oliverio M, Boitani L. Body mass as a predictive variable of home-range size among Italian mammals and birds. *J Zool* 2006; **269**: 317–30.

22 Lislevand T, Figuerola J, Szekely T. Avian Body Sizes in Relation to Fecundity, Mating System, Display Behaviour, and Resource Sharing. *Ecology* 2007; **88**: 8888.

23 Zuur AF, Ieno EN, Elphick CS. A protocol for data exploration to avoid common statistical problems. *Methods Ecol Evol* 2010; **1**: 3–14.

24 Goldstein H, Browne Wi, Rasbash J. Partitioning Variation in Multilevel Models. *Underst Stat* 2002; **1**: 223–31.

25 Wood SN. Fast stable REML and ML estimation of semiparametric GLMs. *J R Stat Soc Ser B (Statistical Methodol* 2011; **73**: 3–36.

26 Pebesma EJ. Multivariable geostatistics in S: the gstat package. *Comput Geosci* 2004; **30**: 683–91.

27 Legendre P, Gauthier O. Statistical methods for temporal and space-time analysis of community composition data. *Proc Biol Sci* 2014; **281**: 20132728.

28 Borcard D, Legendre P, Avois-Jacquet C, Tuomisto H. Dissecting the spatial structure of ecological data at multiple scales. *Ecology* 2004; **85**: 1826–32.
